# Analytical solutions for the Noyes Field model of the time fractional Belousov Zhabotinsky reaction using a hybrid integral transform technique

**DOI:** 10.1038/s41598-024-74072-6

**Published:** 2024-10-23

**Authors:** K. Aruna, N. I. Okposo, K. Raghavendar, Mustafa Inc

**Affiliations:** 1 Department of Mathematics, School of Advanced Sciences, Vellore Institute of Technology, Vellore, Tamil Nadu 632014 India; 2 Department of Mathematics, Delta State University, PMB 1, Abraka, Delta State Nigeria; 3 Department of Mathematics, Firat University, 23119 Elazig, Turkey

**Keywords:** Belousov–Zhabotinsky reaction model, Caputo, Caputo–Fabrizio, Atangana–Baleanu, Natural transform decomposition method, Mathematics and computing, Applied mathematics

## Abstract

In this work, we employed an attractive hybrid integral transform technique known as the natural transform decomposition method (NTDM) to investigate analytical solutions for the Noyes-Field (NF) model of the time-fractional Belousov–Zhabotinsky (TF-BZ) reaction system. The aforementioned time-fractional model is considered within the framework of the Caputo, Caputo–Fabrizio, and Atangana–Baleanu fractional derivatives. The NTDM couples the Adomian decomposition method and the natural transform method to generate rapidly convergent series-type solutions via an elegant iterative approach. The existence and uniqueness of solutions for the considered time-fractional model are first investigated via a fixed-point approach. The reliability and efficiency of the considered solution method is then demonstrated for two test cases of the TF-BZ reaction system. To demonstrate the validity and accuracy of the considered technique, numerical results with respect to each of the mentioned fractional derivatives are presented and compared with the exact solutions as well as with those from existing related literature. Graphical representations depicting the dynamic behaviors of the chemical wave profiles of the concentrations of the intermediates are presented with respect to varying fractional parameter values as well as temporal and spatial variables. The obtained results indicate that the execution of the method is straightforward and can be employed to explore nonlinear time-fractional systems modeling complex chemical reactions.

## Introduction

Many chemical reactions encountered in laboratory experiments are reversible if concentrations of the reactants and products attain equilibrium or irreversible in the case of total conversion of the reactants into products^1^. However, researchers have also observed the existence of another class of chemical reactions characterized by periodic and/or repeated oscillations under certain conditions^2^. This class of chemical reactions (sometimes called chemical oscillators) was once deemed impossible or unrealistic by the chemical scientific community as it negates the equilibrium principle of the second law of thermodynamics^3^. Chemical oscillators are very important to both theoretical and experimental chemists as they demonstrate exceptional cases where that do not exhibit thermodynamic equilibrium. Additionally, they provide evidence that the state of non-equilibrium can persist for an extended length of time with inherent chaotic evolution. It is now well documented that not only does the aforementioned class of chemical reactions exhibit observable wave-like oscillations, it also undergoes periodic variations which depends on the concentrations of one or more of the reacting species^4^. Some members of this class include the Belousov–Zhabotinsky (BZ) reaction, Briggs–Rauscher reaction, and Bray–Liebhafsky reaction^4,5^. Among these, the BZ reaction is one of the most fascinating, well-studied, and well-understood prototype chemical reactions that exhibit self-sustaining oscillatory characteristics under varying conditions^5^. In the early 1950s, Russian biochemist Belousov^6^ made the unintentional discovery of what is now called the BZ reaction while searching for a non-organic counterpart of the Krebs cycle. With a monotonic color transition from colorless Ce(III) ions to pale yellow Ce(IV) ions as an expected result from his test tube mixture of bromate ion ($\text {BrO}_{3}^{-}$) as an oxidizing agent, citric acid as an organic substrate and cerium ions as catalyst, Belousov observed with surprise that the reaction did not proceed to equilibrium methodically and uniformly, like most chemical reactions. Instead, it turned yellow then colorless, then yellow again and colorless, oscillating periodically between oxidized and reduced states. At that time, skeptical journal editors contemptuously turned down the publication of his results even though he incorporated a simple recipe and oscillographic representations of different reaction phases in one of his submissions^3^. His work later gained considerable research interest and was posthumously published in 1981 after the immense research efforts of Anatoly Zhabotinsky^5^. By replacing citric acid and cerium with malonic acid ($\text {CH}_{{2}}$(CO2H)_2_) and ferroin, respectively, Zhabotinsky demonstrated the appearance of cycles of self-sustaining oscillations in the concentrations of the intermediates with visible color change that alternate between bright blue and reddish purple. In the years that followed, the BZ reaction attracted substantial research studies from both theoretical and experimental scientists with a flurry of papers discussing both temporal oscillations and spatial patterns exhibited by the BZ reaction system^7,8^.

While the whole chemical kinetics of the BZ reaction entails several reaction processes with many intermediate steps, it essentially involves a complex Ce(IV)/Ce(III) catalyzed oxidation and bromination of $$\hbox {CH}_{{2}}$$(COOH)_2_ by $$\hbox {BrO}_{3}^{-}$$ in a sulfuric acid ($$\hbox {H}_{{2}}$$$$\hbox {SO}_{{4}}$$) medium^9^. On the the basis of the Field, Körös and Noyes^7^ reaction mechanism for the BZ reaction, Field and Noyes abstracted a simplified five-step reaction model which still retains the significant features of the complete BZ system and which can be approximated by the sequence^8,9^:

1 $$\begin{aligned} \left\{ \begin{aligned}&A+\Phi {\mathop {\longrightarrow }\limits ^{k_{1}}} \Psi +P, \quad \Phi +\Psi {\mathop {\longrightarrow }\limits ^{k_{2}}} P, \\&B+\Psi {\mathop {\longrightarrow }\limits ^{k_{3}}} 2\Phi +Z, \quad 2\Phi {\mathop {\longrightarrow }\limits ^{k_{4}}} Q, \quad Z{\mathop {\longrightarrow }\limits ^{k_{5}}} f\Psi , \end{aligned}\right. \end{aligned}$$

where $k_{1},\cdots ,k_{5}$ are forward reaction rate constants, $\Phi =HBrO _{2}, \ \Psi =Br ^{-}, \ Z=Ce ^{+}_{4}, \ A=BrO ^{-}_{3}, \ P=HOBr$, and *f* is a constant stoichiometric factor^9^. An application of the law of mass action on 1 led to the following system of differential equations:

2 $$\begin{aligned} \left\{ \begin{aligned}&\Phi _{\tau }=k_{1}A\Psi -k_{2}\Phi \Psi +k_{3}B\Phi -k_{4}\Phi ^{2}, \\&\Psi _{\tau }=-k_{1}A\Psi -k_{2}\Phi \Psi +k_{5}fZ, \\&Z_{\tau }=k_{3}B\Phi -k_{5}Z. \end{aligned}\right. \end{aligned}$$

By setting $Z = 0$ in 2 and assuming that the intermediates $\Phi$ and $\Psi$ diffuse with constant diffusion rated $D_{1}$ and $D_{2}$, respectively, Field and Noyes^7,8^ further propose the following reaction-diffusion system to model the BZ reaction system:

3 $$\begin{aligned} \left\{ \begin{aligned}&\Phi _{\tau }=k_{1}A\Psi -k_{2}\Phi \Psi +k_{3}B\Phi -k_{4}\Phi ^{2}+D_1\Phi _{\chi \chi }, \\&\Psi _{\tau }=-k_{1}A\Psi -k_{2}\Phi \Psi +D_2\Psi _{\chi \chi }, \end{aligned}\right. \end{aligned}$$

where $D_{i}>0 \ (i=1,2)$ denotes the diffusion constant and $\chi$ is the spatial coordinate in one-dimension.

One significant feature of the BZ reaction system lies in the fact that it permits visible observation of complex periodic spatio-temporal pattern formations in a time frame of tens of secs and a spatial dimension of a few millimeters. This peculiar characteristics provides significant avenue for extensive scientific investigations into the realm of bifurcation analysis and chaotic oscillatory behaviors of chemical oscillators within the framework of fractional calculus. As a generalized extension of the classical (or integer-order) differential operators to their fractional (or non-integer) order counterparts, fractional calculus has recently attracted extensive interest in diverse research directions. Its theory generally covers all studies related to the properties and analysis of ODEs/PDEs whose derivatives are of fractional order (see for instance^10–28,30^ and the references therein). In existing literature, there are different definitions for different types of fractional order derivatives (FODs). However, the Caputo^11^, Caputo-Fabrizio $\mathtt {(CF)}$^12,13^ and Atangana-Baleanu $\mathtt {(AB)}$^14^ fractional derivatives are the most commonly used types of FODs. While the Caputo derivative is known to employ the power-type kernel with its associated singularity as a limitations, the $\texttt{CF}$ and $\texttt{AB}$ derivatives employ non-singular kernels of exponential and Mittag-Leffler types, respectively^15,16^. Their wide applicability in modern scientific research extends to diverse areas such as mathematical epidemiology^17–21^, climate change^22^, oscillatory systems and electric networks^23^, nanofluid flow^24^, chaotic systems with singular and non-singular kernels^25^, bifurcation of neural network systems with delays^26,27^, catalyzed hydrogenolysis of glycerol^28^, propagation of waves in complex media^29^ and nonlinear optical fibers^30^.

The choice of studying mathematical models within the framework of fractional calculus stems from the fact that the dynamics of many physical processes incorporate intricate nonlocality behaviors which cannot be adequately explained within the context of classical calculus^31,32^. Many studies have demonstrated that the nonlocality behaviors incorporate a variety of complex global attributes such as non-Markovian processes, fractal behaviors, stochastic and anomalous diffusion processes, random walk as well as memory and historical effects^33^. Unlike the local properties associated with integer order differential operators, the nonlocal behaviors of FODs allow that the future states of any physical system are not only influenced by the current state, but also by all other preceding states^10^. From this perspective, nonlocal behaviors characterizing the chaotic and non-equilibrium phenomena of the BZ reaction system are evidenced by periodic oscillations between oxidized and reduced states of the intermediates. These periodic oscillations which are observable via color transitions under experimental settings are further affirmation of the influence of memory effects where past interactions of the intermediates depend on their future states. Furthermore, in view of the the high degree of freedom of FODs, the ability of chemical oscillators to produce thousands of oscillatory cycles within a closed system allows for significant studies and numerical simulations of a wide range of chemical waves profiles and patterns without any need for a constant replenishment of the reacting species.

The focus of this work is to obtain and investigate analytical solutions of the following non-dimensionalized Noyes-Field model for the time-fractional Belousov-Zhabotinsky (TF-BZ) reaction^34–36^:

4 $$\begin{aligned} \left\{ \begin{aligned}&^{{\Xi (\mu )}}\textrm{D}_{\tau }^{\mu }\Phi =\varrho _{1}\Phi _{\chi \chi }+\beta \xi \Psi +\Phi (1-\Phi -\xi \Psi ),\\&^{{\Xi (\mu )}}\textrm{D}_{\tau }^{\mu }\Psi =\varrho _{2}\Psi _{\chi \chi }+\gamma \Psi -\zeta \Phi \Psi , \\&\Phi (\chi ,0)=h_{1}(\chi ), \Psi (\chi ,0)=h_{2}(\chi ). \end{aligned} \right. \end{aligned}$$

Here, $^{{\Xi (\mu )}}\textrm{D}^{\mu }_{\tau }$ denotes the fractional derivative either in the Caputo, $\texttt{CF}$ or $\texttt{AB}$ sense and $\mu \in (0,1]$ is the fractional parameter index. In view of 1–3, $\Phi =\Phi (\chi ,\tau )$ and $\Psi =\Psi (\chi ,\tau )$ are space-time dependent functions denoting concentrations of bromous acid ($\hbox {HBrO}_{{2}}$) and bromide ion ($\hbox {Br}^{-}$) with respective diffusion coefficients $\varrho _{1}$ and $\varrho _{2}$. Additionally, $\gamma$ and $\beta$ are constants while $\xi$ and $\zeta \ne 1$ are positive parameters. Although, there exists no known direct method for finding closed-form solutions of nonlinear fractional order PDEs, numerous semi-analytical and numerical techniques have been effectively employed to obtain approximate solutions for this class of problems. Particularly, on studies related to the BZ reaction system, Akinyemi^34^ and Baishya and Veeresha^35^ employed the $q-$homotopy analysis transform method and Laplace transform to obtain numerical solution for the TF-BZ reaction system with Caputo derivative. Karaagac et al.^36^ considered the TF-BZ model with $\texttt{AB}$ fractional derivative by using the fractional version of the Adams-Bashforth technique. Algehyne et. al^37^ employed a hybrid technique that combines the power series approach and Lie symmetry method to extract analytical solutions for the classical model. Alaoui et al.^38^ investigated solutions of the TF-BZ model by using the Yang transform decomposition method (YTDM) and the homotopy perturbation Yang transform method (HPYTM). An adapted Runge-Kutta method was employed in^39^ to study the classical BZ system. Alsallami et. al^40^ applied the double Laplace transform method to study the TF-BZ system with respect to the Caputo and $\texttt{AB}$ fractional derivatives. Recently, Rehman et. al^41^ studied the approximate solutions of TF-BZ system by using natural transform iterative method (NTIM) and optimal homotopy asymptotic method (OHAM). Other methods that have also been used to obtain approximate solutions for other nonlinear fractional order PDEs include the differential transform method^42^, homotopy perturbation method and its hybrid versions^43,44^, Elzaki and Yang transform methods as well as their hybrid versions^45^, homotopy Sumudu transform method^46^, homotopy analysis method and its hybrid versions^47^ and the natural transform decomposition method (NTDM)^48^.

With the aim of investigating the effects of varying fractional order index $\mu$ on the chemical wave profiles for the concentration of the intermediates, this work employs the NTDM to derive analytic solutions for the TF-BZ reaction system 4. In contrast to other methods, the NTDM is a hybrid integral transform technique that elegantly incorporates the Adomian polynomial into the natural transform method (NTM) to provide a simplified iterative scheme that ensures rapid convergence of the generated series. This method does not incorporate massive calculations or round-off errors. It has great accuracy with minimal processing time. The NTDM is easy to implement and is notable for producing suitable approximate solutions for both linear and nonlinear fractional differential equations. To the best of our knowledge, the obtained results are compared for the Caputo, $\texttt{CF}$ and $\texttt{AB}$ FODs for the first time in the same study as well as with results obtained for the exact solutions and from existing literature.

The rest part of this paper is highlighted as follows: section "Mathematical preliminaries" provides important preliminary tools needed for subsequent sections of this work. Section "Some qualitative analysis of the TF-BZ reaction system" presents some useful qualitative features on the solution of the TF-BZ reaction system (4) as well as the result on the existence of a unique solution via fixed point theory. The NTDM solution procedure is discussed in section "NTDM solution procedure" for a general coupled system of time-fractional differential equations. In section "Numerical implementation and results" the NTDM is applied to the following two cases of the TF-BZ reaction system. For each of the considered cases, we investigate the obtained numerical results with respect to the Caputo $\texttt{CF}$ and $\texttt{AB}$ derivatives. Section "Discussion of results" presents a discussion on the obtained results while in section "Conclusion" a conclusion is provided.

## Mathematical preliminaries

The Caputo, $\texttt{CF}$ and $\texttt{AB}$ derivatives have been the most extensively used fractional derivatives in recent times. On the nature of their kernels, the Caputo derivative has a power-type kernel function, whereas the $\texttt{CF}$ and $\texttt{AB}$ derivatives have the exponential and Mittag-Leffler kernel functions, respectively. This section presents some important information on the above-mentioned derivatives as well as the natural transform operator.

### Definition 1

^11^The Riemann-Liouville fractional integral of order $\mu$ of a function $g\in C_{\mu }, \ \mu \ge -1$ is defined as

5 $$\begin{aligned} \mathcal {J}^{\mu }_{\tau }\Phi (\chi ,\tau )= \left\{ \begin{aligned}&\Phi (\chi ,\tau ),&\quad&\mu =0, \ \tau>0,\\&\frac{1}{\Gamma (\mu )}\int _{0}^{\tau }(\tau -\varpi )^{\mu -1}\Phi (\chi ,\varpi )d\varpi ,&\quad&\mu>0, \ \tau >0. \end{aligned}\right. \end{aligned}$$

### Definition 2

^11^For a continuous function $\Phi (\chi ,\tau )$, the Caputo fractional partial derivative (FPD) is given as

6 $$\begin{aligned} ^{\texttt{C}}\textrm{D}^{\mu }_{\tau }\Phi (\chi ,\tau )= \frac{1}{\Gamma (\kappa -\mu )}\int _{0}^{\tau }(\tau -\varpi )^{\kappa -\mu -1}\Phi ^{(\kappa )}(\chi ,\varpi )d\varpi , \ \ \kappa -1<\mu \le \kappa \in \mathbb {N}, \end{aligned}$$

and

7 $$\begin{aligned} ^{\texttt{C}}\textrm{D}^{\mu }_{\tau }\Phi (\chi ,\tau )=\frac{\partial ^{\kappa }\Phi (\chi ,\tau )}{\partial \tau ^{\kappa }}, \ \ \mu =\kappa \in \mathbb {N}. \end{aligned}$$

If $$\kappa =1$$^6^ becomes

8 $$\begin{aligned} ^{\texttt{C}}\textrm{D}^{\mu }_{\tau }\Phi (\chi ,\tau )= \frac{1}{\Gamma (1-\mu )}\int _{0}^{\tau }(\tau -\varpi )^{-\mu }\Phi '(\chi ,\varpi )d\varpi , \ \ 0<\mu \le 1\in \mathbb {N}. \end{aligned}$$

### Definition 3

^12^Let $\tau >0$ and $\mu \in (0,1]$. The $\texttt{CF}$ FPD of a function $\Phi (\chi ,\tau )$ is given as

9 $$\begin{aligned} ^{\texttt{CF}}\textrm{D}^{\mu }_{\tau }\Phi (\chi ,\tau )=\frac{\mathcal {M}(\mu )}{1-\mu } \int _{0}^{\tau }\exp \left[ -\frac{\mu }{1-\mu }(\tau -\varpi )\right] \Phi '(\chi ,\varpi )d\varpi , \end{aligned}$$

where $\mathcal {M}(\mu )$ denotes the normalization functions satisfying $\mathcal {M}(0)=\mathcal {M}(1)=1$.

### Definition 4

^13^The definition of the fractional integral associated with the $\texttt{CF}$ derivative is

10 $$\begin{aligned} ^{\texttt{CF}}\mathcal {J}^{\mu }_{\tau }\Phi (\chi ,\tau )=\frac{1-\mu }{\mathcal {M}(\mu )} \Phi (\chi ,\tau )+\frac{\mu }{\mathcal {M}(\mu )}\int _{0}^{\tau } \Phi (\chi ,\varpi )d\varpi , \ \ 0<\mu <1, \ \ \tau \ge 0. \end{aligned}$$

### Definition 5

^14^Let $\tau >0$ and $\mu \in (0,1]$. The $\texttt{AB}$ FPD of a function $\Phi (\chi ,\tau )$ is given as

11 $$\begin{aligned} ^{\texttt{AB}}\textrm{D}^{\mu }_{\tau }\Phi (\chi ,\tau )=\frac{\mathcal {B}(\mu )}{1-\mu }\int _{0}^{\tau }\mathbb {E}_{\mu }\left[ -\frac{\mu }{1-\mu }(\tau -\varpi )^{\mu }\right] \Phi '(\chi ,\varpi )d\varpi , \end{aligned}$$

where $\mathbb {E}_{\mu }[\cdot ]$ denotes the Mittag-Leffler function and $\mathcal {B}(\mu )$ is the normalization functions satisfying the same property $\mathcal {B}(0)=\mathcal {B}(1)=1$.

### Definition 6

^14^The fractional integral related to the $\texttt{AB}$ derivative is defined as

12 $$\begin{aligned} ^{\texttt{AB}}\mathcal {J}^{\mu }_{\tau }\Phi (\chi ,\tau )=\frac{1-\mu }{\mathcal {B}(\mu )} \Phi (\chi ,\tau )+\frac{\mu }{\mathcal {B}(\mu )\Gamma (\mu )} \int _{0}^{\tau }(\tau -\varpi )^{\mu -1}\Phi (\chi ,\tau )d\varpi . \end{aligned}$$

### Definition 7

^49–52^The natural transform $\mathbb{N}\mathbb{T}[\cdot ]$ is defined over the set of functions

$$\mathbb {S}=\left\{ \Phi (\chi ,\tau ): \exists \ K, r_{1},r_{2} >0, |\Phi (\chi ,\tau )|<K\exp \left( \frac{|t|}{r_{i}}\right) , \ if \ \tau \in (-1)^{i}\times [0,\infty ) \right\} ,$$

by the integral

13 $$\begin{aligned} \mathbb{N}\mathbb{T}[\Phi (\chi ,\tau )]=\int _{-\infty }^{\infty }\exp (-s\tau )\Phi (\chi ,\omega \tau )d\tau , \ \ -\infty<s,\omega <\infty , \end{aligned}$$

and for $\tau \in (0,\infty )$ the natural transform is defined by the integral

14 $$\begin{aligned} \mathbb{N}\mathbb{T}^{+}[\Phi (\chi ,\tau )]=\int _{0}^{\infty }\exp (-s\tau )\Phi (\chi ,\omega \tau )d\tau , \ \ 0<s,\omega <\infty , \end{aligned}$$

where *s* and $\omega$ are the natural transform parameters.

### Definition 8

For the function $\Phi (\chi ,s,\omega )$, the inverse natural transformation is given as

15 $$\begin{aligned} \mathbb{N}\mathbb{T}^{-1}[\Phi (\chi ,s,\omega )]=\Phi (\chi ,\tau ), \ \forall \ \tau \ge 0. \end{aligned}$$

Furthermore, the following important properties are satisfied by the natural transform operator^49–52^:

(i) $$\mathbb{N}\mathbb{T}^{+}[1]=\displaystyle {\frac{1}{s}}; \ \ \mathbb{N}\mathbb{T}^{+}[t]=\displaystyle {\frac{\omega }{s^{2}}};$$
(ii) $$\mathbb{N}\mathbb{T}^{+}\left[ \displaystyle {\frac{\tau ^{\kappa -1}}{(\kappa -1)!}}\right] =\displaystyle {\frac{\omega ^{\kappa -1}}{s^{\kappa }}}, \kappa =1,2,\cdots ; \ \ \mathbb{N}\mathbb{T}^{+}[\tau ^{\mu }]=\displaystyle {\frac{\mu (\mu +1)\omega ^{\mu }}{s^{\mu +1}}}, \ \mu >-1;$$
(iii) $\mathbb{N}\mathbb{T}^{+}[\delta _{1}\Phi _{1}(\chi ,\tau )+\delta _{2}\Phi _{2}(\chi ,\tau )]=\delta _{1}\mathbb{N}\mathbb{T}^{+}[\Phi _{1}(\chi ,\tau )]+\delta _{2}\mathbb{N}\mathbb{T}^{+}[\Phi _{2}(\chi ,\tau )]$ where $\delta _{1}$ and $\delta _{2}$ are positive constants;
(iv) $$\mathbb{N}\mathbb{T}^{+}[\Phi ^{\kappa }(\chi ,\tau )]=\displaystyle {\left( \frac{s}{\omega }\right) ^{\kappa }\mathbb{N}\mathbb{T}^{+}[\Phi (\chi ,\tau )]}-\sum _{k=0}^{\kappa -1}\frac{s^{\kappa -k-1}}{\omega ^{\kappa -k}}\Phi ^{(k)}(\chi ,0), \ \kappa \in \mathbb {N}$$.

### Theorem 1

^49–52^Let $\mathbb{N}\mathbb{T}^{+}[\Phi (\chi ,\tau )]$ denote the natural transform of the function $\Phi (\chi ,\tau )$. Then the natural transform for the Caputo, $\texttt{CF}$ and $\texttt{AB}$ derivatives of $\Phi (\chi ,\tau )$ are given by

16 $$\begin{aligned} & \mathbb{N}\mathbb{T}^{+}\left[ ^{\texttt{C}}\textrm{D}^{\mu }_{\tau }\Phi (\chi ,\tau )\right] = \left( \frac{s}{\omega }\right) ^{\mu }\left[ \mathbb{N}\mathbb{T}^{+}[\Phi (\chi ,\tau )]-\left( \frac{1}{s}\right) \Phi (\chi ,0)\right] , \end{aligned}$$

17 $$\begin{aligned} & \mathbb{N}\mathbb{T}^{+}\left[ ^{\texttt{CF}}\textrm{D}^{\mu }_{\tau }\Phi (\chi ,\tau )\right] = \frac{1}{1-\mu +\mu \left( \omega /s\right) }\left[ \mathbb{N}\mathbb{T}^{+}[\Phi (\chi ,\tau )]-\left( \frac{1}{s}\right) \Phi (\chi ,0)\right] , \end{aligned}$$

and

18 $$\begin{aligned} \mathbb{N}\mathbb{T}^{+}\left[ ^{\texttt{AB}}\textrm{D}^{\mu }_{\tau }\Phi (\chi ,\tau )\right] = \frac{\mathcal {B}(\mu )}{1-\mu +\mu \left( \omega /s\right) ^{\mu }}\left[ \mathbb{N}\mathbb{T}^{+}[\Phi (\chi ,\tau )]-\left( \frac{1}{s}\right) \Phi (\chi ,0)\right] , \end{aligned}$$

respectively.

## Some qualitative analysis of the TF-BZ reaction system

This section presents a qualitative analysis of the TF-BZ reaction system (4). Firstly we establish some estimates and then proceed to investigate the existence of a unique solution to the considered problem. In this direction, let

19 $$\begin{aligned} \mathcal {Z}_{1}(\chi ,\tau ,\Phi ,\Psi )=\varrho _{1}\Phi _{\chi \chi }+\beta \xi \Psi +\Phi (1-\Phi +\xi \Psi ) \ \ \text{ and } \ \ \mathcal {Z}_{2}(\chi ,\tau ,\Phi ,\Psi )=\varrho _{2}\Psi _{\chi \chi }+\gamma \Psi -\zeta \Phi \Psi , \end{aligned}$$

to represent the right-hand terms of each equation of (4), the TF-BZ system can be rewritten as

20 $$\begin{aligned} \left\{ \begin{aligned}&^{{\Xi (\mu )}}\textrm{D}^{\mu }_{\tau }\Phi (\chi ,\tau )=\mathcal {Z}_{1}(\chi ,\tau ,\Phi ,\Psi ),\\&^{{\Xi (\mu )}}\textrm{D}^{\mu }_{\tau }\Psi (\chi ,\tau )=\mathcal {Z}_{2}(\chi ,\tau ,\Phi ,\Psi ), \end{aligned}\right. \end{aligned}$$

so that the existence and uniqueness of its solutions can be investigated under certain conditions via a fixed-point approach. To this end, let $\Phi$ and $\Psi$ be bounded above and $\Vert (\Phi -\bar{\Phi })_{\chi \chi }\Vert \le \delta _{1}\Vert \Phi -\bar{\Phi }\Vert$, $\Vert (\Psi -\bar{\Psi })_{\chi \chi }\Vert \le \delta _{2}\Vert \Psi -\bar{\Psi }\Vert$, $\Vert \Phi \Vert \le m_{1}$, $\Vert \Psi \Vert \le m_{1}$ for some constants $\delta _{1},\delta _{1},m_{1}, m_{2}>0$. Then for any pair of solutions $(\Phi ,\Psi )$ and $(\bar{\Phi },\bar{\Psi })$ of (4) the triangle inequality yield

21 $$\begin{aligned} \Vert \mathcal {Z}_{1}(\chi ,\tau ,\Phi ,\Psi )-\mathcal {Z}_{1}(\chi ,\tau ,\bar{\Phi },\bar{\Psi })\Vert \le \mathcal {L}_{1}\Vert \Phi -\bar{\Phi }\Vert \ \ \text{ and } \ \ \Vert \mathcal {Z}_{2}(\chi ,\tau ,\Phi ,\Psi )-\mathcal {Z}_{2}(\chi ,\tau ,\bar{\Phi },\bar{\Psi })\Vert \le \mathcal {L}_{2}\Vert \Psi -\bar{\Psi }\Vert , \end{aligned}$$

where $\mathcal {L}_{1}:=(\varrho _{1}\delta _{1}+1-m_{1}+\xi m_{2})> 0$ and $\mathcal {L}_{2}:=(\varrho _{2}\delta _{2}+\gamma +m_{1})> 0$. Moreover, if $0\le \mathcal {L}_{1},\mathcal {L}_{2}<1$, then the nonlinear functions are $\mathcal {Z}_{1}(\chi ,\tau ,\Phi ,\Psi )$ ans $\mathcal {Z}_{2}(\chi ,\tau ,\Phi ,\Psi )$ as well contractions.

### Theorem 2

Consider the time-fractional BZ reaction model (4) in the sense of Caputo. Then under the assumption that $\Phi (\chi ,\tau )$ and $\Psi (\chi ,\tau )$ are bounded functions, the operators $\mathbb {T}[\Phi (\chi ,\tau )]$ and $\mathbb {T}[\Psi (\chi ,\tau )]$ expressed as

22 $$\begin{aligned} \mathbb {T}[\Phi (\chi ,\tau )]=\Phi (\chi ,0)+\frac{1}{\Gamma (\mu )}\int _{0}^{\tau } \mathcal {Z}_{1}(\chi ,\varpi ,\Phi ,\Psi )(\tau -\varpi )^{\mu -1}d\varpi , \end{aligned}$$

and

23 $$\begin{aligned} \mathbb {T}[\Psi (\chi ,\tau )]=\Psi (\chi ,0)+\frac{1}{\Gamma (\mu )}\int _{0}^{\tau } \mathcal {Z}_{2}(\chi ,\varpi ,\Phi ,\Psi ) (\tau -\varpi )^{\mu -1}d\varpi , \end{aligned}$$

respectively, satisfy the Lipschitz condition (LC).

### Proof

Given limited functions $\Phi _{1}(\chi ,0)=\Phi _{2}(\chi ,0)$, let $\Phi _{1}(\chi ,\tau )$ and $\Psi _{2}(\chi ,\tau )$ be defined. Then

$$\begin{aligned} \begin{aligned}&\Vert \mathbb {T}[\Phi _{1}(\chi ,\tau )]-\Psi [\Phi _{2}(\chi ,\tau )]\Vert \\&\le \frac{1}{\Gamma (\mu )} \int _{0}^{\tau }\Vert \mathcal {Z}_{1}(\chi ,\varpi ,\Phi ,_{1},\Psi _{1}) -\mathcal {Z}_{1}(\chi ,\varpi ,\Phi ,_{2},\Psi _{1})\Vert (\tau -\varpi )^{\mu -1}d\varpi \le \frac{\tau ^{\mu }}{\Gamma (\mu +1)}\mathcal {L}_{1}\Vert \Phi _{1}-\Phi _{2}\Vert . \end{aligned} \end{aligned}$$

This implies

$$\begin{aligned} \Vert \mathbb {T}[\Phi _{1}(\chi ,\tau )]-\Psi [\Phi _{2}(\chi ,\tau )]\Vert \le \mathscr {Z}_{1}\Vert \Phi _{1}-\Phi _{2}\Vert , \end{aligned}$$

where $\mathscr {Z}_{1}:=\frac{\tau ^{\mu }}{\Gamma (\mu +1)}\mathcal {L}_{1}$. Similarly, we have

$$\begin{aligned} \begin{aligned} \Vert \mathbb {T}[\Psi _{1}(\chi ,\tau )]-\Psi [\Psi _{2}(\chi ,\tau )]\Vert \le \mathscr {Z}_{2}\Vert \Psi _{1}-\Psi _{2}\Vert , \end{aligned} \end{aligned}$$

for any two bounded functions $\Psi _{1}(\chi ,\tau )$ and $\Psi _{2}(\chi ,\tau )$ with $\Psi _{1}(\chi ,0)=\Psi _{2}(\chi ,0)$, where $\mathscr {Z}_{2}:=\frac{\tau ^{\mu }}{\Gamma (\mu +1)}\mathcal {L}_{2}$. $\square$

### Theorem 3

Let the time-fractional differential operator in (4) be defined in the $\texttt{CF}$ sense. Then under the assumption that $\Phi (\chi ,\tau )$ and $\Psi (\chi ,\tau )$ are bounded functions, the operators $\mathbb {T}[\Phi (\chi ,\tau )]$ and $\mathbb {T}[\Psi (\chi ,\tau )]$ expressed as

24 $$\begin{aligned} \mathbb {T}[\Phi (\chi ,\tau )]=\Phi (\chi ,0)+\frac{(1-\mu )}{\mathcal {M}(\mu )} \mathcal {Z}_{1}(\chi ,\varpi ,\Phi ,\Psi )+\frac{\mu }{\mathcal {M}(\mu )}\int _{0}^{\tau } \mathcal {Z}_{1}(\chi ,\varpi ,\Phi ,\Psi )(\tau -\varpi )^{\mu -1}d\varpi , \end{aligned}$$

and

25 $$\begin{aligned} \mathbb {T}[\Psi (\chi ,\tau )]=\Psi (\chi ,0)+\frac{(1-\mu )}{\mathcal {M}(\mu )} \mathcal {Z}_{2}(\chi ,\varpi ,\Phi ,\Psi )+\frac{\mu }{\mathcal {M}(\mu )}\int _{0}^{\tau } \mathcal {Z}_{2}(\chi ,\varpi ,\Phi ,\Psi ) (\tau -\varpi )^{\mu -1}d\varpi , \end{aligned}$$

respectively, satisfy the LC.

### Proof

The proof is similar to that of Theorem 2 and is therefore omitted. $\square$

### Theorem 4

Let the time-fractional differential operator in (4) be defined in the $\texttt{AB}$ sense. Then under the assumption that $\Phi (\chi ,\tau )$ and $\Psi (\chi ,\tau )$ are bounded functions, the operators $\mathbb {T}[\Phi (\chi ,\tau )]$ and $\mathbb {T}[\Psi (\chi ,\tau )]$ expressed as

26 $$\begin{aligned} \mathbb {T}[\Phi (\chi ,\tau )]=\Phi (\chi ,0)+\frac{(1-\mu )}{\mathcal {B}(\mu )} \mathcal {Z}_{1}(\chi ,\varpi ,\Phi ,\Psi )+\frac{\mu }{\Gamma (\mu )\mathcal {B}(\mu )} \int _{0}^{\tau }\mathcal {Z}_{1}(\chi ,\varpi ,\Phi ,\Psi )(\tau -\varpi )^{\mu -1}d\varpi , \end{aligned}$$

and

27 $$\begin{aligned} \mathbb {T}[\Psi (\chi ,\tau )]=\Psi (\chi ,0)+\frac{(1-\mu )}{\mathcal {B}(\mu )} \mathcal {Z}_{2}(\chi ,\varpi ,\Phi ,\Psi )+\frac{\mu }{\Gamma (\mu )\mathcal {M}(\mu )}\int _{0}^{\tau } \mathcal {Z}_{2}(\chi ,\varpi ,\Phi ,\Psi ) (\tau -\varpi )^{\mu -1}d\varpi , \end{aligned}$$

respectively, satisfy the LC.

### Proof

The proof is similar to that of Theorem 2 and is therefore omitted. $\square$

### Theorem 5

Suppose that $\Phi (\chi ,\tau )$ and $\Psi (\chi ,\tau )$ are bounded functions, then the operators given by

$$\mathbb {F}(\Phi )=\varrho _{1}\Phi _{\chi \chi }+\beta \xi \Psi +\Phi (1-\Phi +\xi \Psi ) \ \ \text{ and } \ \ \mathbb {F}(\Psi )=\varrho _{2}\Psi _{\chi \chi }+\gamma \Psi -\zeta \Phi \Psi ,$$

satisfy $$|\left<\mathbb {F}(\Phi )-\mathbb {F}(\Phi _{1}),\Phi -\Phi _{1}\right>|\le \mathscr {S}_{1}\Vert \Phi -\Phi _{1}\Vert ^{2} \ \ \text{ and } \ \ |\left<\mathbb {F}(\Psi )-\mathbb {F}(\Psi _{1}),\Psi -\Psi _{1}\right>|\le \mathscr {S}_{2}\Vert \Psi -\Psi _{1}\Vert ^{2},$$ respectively.

### Proof

Let $\Phi (\chi ,\tau )$ and $\Psi (\chi ,\tau )$ be bounded functions, then we get

$$\begin{aligned} \begin{aligned} |\left\langle \mathbb {F}(\Phi )-\mathbb {F}(\Phi _{1}),\Omega \right\rangle |=&\left| \left\langle \left( \Big [\varrho _{1}\Phi _{\chi \chi }+\beta \xi \Psi +\Phi \Big (1-\Phi +\xi \Psi \Big )\Big ] -\Big [\varrho _{1}\Phi _{1xx}+\beta \xi \Psi +\Phi _{1}\Big (1-\Phi _{1}+\xi \Psi \Big )\Big ]\right) ,\Phi -\Phi _{1}\right\rangle \right| \\ \le&\left| \left\langle \varrho _{1}(\Phi -\Phi _{1})_{\chi \chi },\Phi -\Phi _{1}\right\rangle \right| +\left| \left\langle \Phi \Big (1-\Phi +\xi \Psi \Big )-\Phi _{1}\Big (1-\Phi _{1}+\xi \Psi \Big ),\Phi -\Phi _{1}\right\rangle \right| \\ \le&\varrho _{1}\Vert (\Phi -\Phi _{1})_{\chi \chi }\Vert \Vert \Phi -\Phi _{1}\Vert +\Big (1+\Vert \Phi \Vert +\Vert \Phi _{1}\Vert +\xi \Vert \Psi \Vert \Big )\Vert \Phi -\Phi _{1}\Vert ^{2}\\ \le&\Big [\varrho _{1}\delta _{1}+2m_{1}+\xi m_{2}\Big ]\Vert \Phi -\Phi _{1}\Vert ^{2}. \end{aligned} \end{aligned}$$

By setting $\mathscr {S}_{1}=\Big [\varrho _{1}\delta _{1}+2m_{1}+\xi m_{2}\Big ]$ we have

$$|\left<\mathbb {F}(\Phi )-\mathbb {F}(\Phi _{1}),\Phi -\Phi _{1}\right>|\le \mathscr {S}_{1}\Vert \Phi -\Phi _{1}\Vert ^{2}.$$

Similarly, we have $|\left<\mathbb {F}(\Psi )-\mathbb {F}(\Psi _{1}),\Psi -\Psi _{1}\right>|\le \mathscr {S}_{2}\Vert \Psi -\Psi _{1}\Vert ^{2}$ where $\mathscr {S}_{2}=\Big [\varrho _{2}\delta _{2}+\gamma +\zeta m_{1}\Big ]$ . This completes the proof. $\square$

### Theorem 6

Let $\Phi (\chi ,\tau )$ and $\Psi (\chi ,\tau )$ be bounded functions and $0<\Vert \Omega \Vert <\infty$ then the operators

$$\mathbb {F}(\Phi )=\varrho _{1}\Phi _{\chi \chi }+\beta \xi \Psi +\Phi (1-\Phi +\xi \Psi ) \ \ \text{ and } \ \ \mathbb {F}(\Psi )=\varrho _{2}\Psi _{\chi \chi }+\gamma \Psi -\zeta \Phi \Psi ,$$

satisfy:

$$|\left<\mathbb {F}(\Phi )-\mathbb {F}(\Phi _{1}),\Omega \right>|\le \mathscr {S}_{1}\Vert \Phi -\Phi _{1}\Vert \Vert \Omega \Vert \ \text{ and } \ |\left<\mathbb {F}(\Psi )-\mathbb {F}(\Psi _{1}),\Omega \right>|\le \mathscr {S}_{2}\Vert \Psi -\Psi _{1}\Vert \Vert \Omega \Vert ,$$

respectively.

### Proof

Let the functions $\Phi (\chi ,\tau )$ and $\Psi (\chi ,\tau )$ be bounded and $0<\Vert \Omega \Vert <\infty$, then we acquire

$$\begin{aligned} \begin{aligned} |\left\langle \mathbb {F}(\Phi )-\mathbb {F}(\Phi _{1}),\Omega \right\rangle |=&\left| \left\langle \left( \Big [\varrho _{1}\Phi _{\chi \chi }+\beta \xi \Psi +\Phi \Big (1-\Phi +\xi \Psi \Big )\Big ] -\Big [\varrho _{1}\Phi _{1xx}+\beta \xi \Psi +\Phi _{1}\Big (1-\Phi _{1}+\xi \Psi \Big )\Big ]\right) ,\Omega \right\rangle \right| \\ \le&\left| \left\langle \varrho _{1}(\Phi -\Phi _{1})_{\chi \chi },\Omega \right\rangle \right| +\left| \left\langle \Phi \Big (1-\Phi +\xi \Psi \Big )-\Phi _{1}\Big (1-\Phi _{1}+\xi \Psi \Big ),\Omega \right\rangle \right| \\ \le&\varrho _{1}\Vert (\Phi -\Phi _{1})_{\chi \chi }\Vert \Vert \Omega \Vert +\Big (1+\Vert \Phi \Vert +\Vert \Phi _{1}\Vert +\xi \Vert \Psi \Vert \Big )\Vert \Phi -\Phi _{1}\Vert \Vert \Omega \Vert \\ \le&\mathscr {S}_{1}\Vert \Phi -\Phi _{1}\Vert \Vert \Omega \Vert . \end{aligned} \end{aligned}$$

Hence,

$$|\left<\mathbb {F}(\Phi )-\mathbb {F}(\Phi _{1}),\Omega \right>|\le \mathscr {S}_{1}\Vert \Phi -\Phi _{1}\Vert \Vert \Omega \Vert ,$$

where $\mathscr {S}_{1}=\Big [\varrho _{1}\delta _{1}+2m_{1}+\xi m_{2}\Big ]$. Similarly, for $\mathscr {S}_{2}=\Big [\varrho _{2}\delta _{2}+\gamma +\zeta m_{1}\Big ]$, it can be shown that

$$\begin{aligned} |\left<\mathbb {F}(\Psi )-\mathbb {F}(\Psi _{1}),\Omega \right>|\le \mathscr {S}_{2}\Vert \Psi -\Psi _{1}\Vert \Vert \Omega \Vert . \end{aligned}$$

This completes the proof. $\square$

### Existence and uniqueness analysis

#### Theorem 7

Let $\Phi (\chi ,\tau )$ and $\Psi (\chi ,\tau )$ be bounded functions, then the solution of the time-fractional IVP (20) unique if

28 $$\begin{aligned} \left\{ \begin{aligned}&\left( 1-\frac{\tau ^{\mu }}{\Gamma (\mu +1)}\mathcal {L}_{i}\right)> 0, & \qquad \text{ if } \ \ ^{{\Xi (\mu )}}\textrm{D}_{\tau }^{\mu }= \ ^{\texttt{C}}\textrm{D}_{\tau }^{\mu },\\&\left( 1-\frac{2(1-\mu )}{(2-\mu )\mathcal {M}(\mu )}\mathcal {L}_{i}-\frac{2\mu }{(2-\mu )\mathcal {M}(\mu )}\mathcal {L}_{i}\tau \right)> 0, & \qquad \text{ if } \ \ ^{{\Xi (\mu )}}\textrm{D}_{\tau }^{\mu }= \ ^{\texttt{CF}}\textrm{D}_{\tau }^{\mu },\\&\left( 1-\frac{1-\mu }{\mathcal {B}(\mu )}\mathcal {L}_{i}-\frac{\tau ^{\mu }}{\mathcal {B}(\mu )\Gamma (\mu )}\mathcal {L}_{i}\right) > 0, & \qquad \text{ if } \ \ ^{{\Xi (\mu )}}\textrm{D}_{\tau }^{\mu }= \ ^{\texttt{AB}}\textrm{D}_{\tau }^{\mu },\\&i=1,2. \end{aligned}\right. \end{aligned}$$

Consequently, the solution of the TF-BZ model (4) is unique.

#### Proof

**Existence:** Application of any of the fractional integral operators (5), (10) or (12) on the time-fractional IVP (20) yields

29 $$\begin{aligned} \left\{ \begin{aligned} \Phi (\chi ,\tau )-\Phi (\chi ,0)&=\mathcal {J}^{\Xi (\mu )}_{\tau }\mathcal {Z}_{1}(\chi ,\varpi ,\Phi ,\Psi ),\\ \Psi (\chi ,\tau )-\Psi (\chi ,0)&=\mathcal {J}^{\Xi (\mu )}_{\tau }\mathcal {Z}_{2}(\chi ,\varpi ,\Phi ,\Psi ), \end{aligned}\right. \end{aligned}$$

where $\mathcal {J}^{\Xi (\mu )}_{\tau }$ denotes the time-fractional integral operator either in the Caputo, $\texttt{CF}$ or $\texttt{AB}$ sense. In particular, if the fractional derivative of (20) is considered in the Caputo sense, then the system of integral equations (29) reads

30 $$\begin{aligned} \left\{ \begin{aligned} \Phi (\chi ,\tau )-\Phi (\chi ,0)&= \frac{1}{\Gamma (\mu )}\int _{0}^{\tau }(\tau -\varpi )^{\mu -1}\mathcal {Z}_{1}(\chi ,\varpi ,\Phi ,\Psi )d\varpi ,\\ \Psi (\chi ,\tau )-\Psi (\chi ,0)&=\frac{1}{\Gamma (\mu )} \int _{0}^{\tau }(\tau -\varpi )^{\mu -1}\mathcal {Z}_{2}(\chi ,\varpi ,\Phi ,\Psi )d\varpi . \end{aligned}\right. \end{aligned}$$

Given the integral representations in (30), the following recursive integral equations are presented:

31 $$\begin{aligned} \left\{ \begin{aligned}&\Phi _{\kappa +1}(\chi ,\tau )=\frac{1}{\Gamma (\mu )}\int _{0}^{\tau } (\tau -\varpi )^{\mu -1}\mathcal {Z}_{1}(\chi ,\varpi ,\Phi ,_{\kappa },\Psi _{\kappa })d\varpi ,\\&\Psi _{\kappa +1}(\chi ,\tau )=\frac{1}{\Gamma (\mu )}\int _{0}^{\tau } (\tau -\varpi )^{\mu -1}\mathcal {Z}_{2}(\chi ,\varpi ,\Phi ,_{\kappa },\Psi _{\kappa })d\varpi ,\\&\kappa =0,1,2,\cdots , \end{aligned}\right. \end{aligned}$$

with initial conditions

32 $$\begin{aligned} \Phi _{0}(\chi ,\tau )=\Phi (\chi ,0)=\Phi _{0}(\chi ), \ \ \Psi _{0}(\chi ,\tau )=\Psi (\chi ,0)=\Psi _{0}(\chi ). \end{aligned}$$

The difference between each integral equations subsequent terms in (31) is therefore taken into consideration as

33 $$\begin{aligned} \left\{ \begin{aligned} \Xi ^{1}_{\kappa }(\chi ,\tau ):=&\Phi _{\kappa }(\chi ,\tau )-\Phi _{\kappa -1}(\chi ,\tau )=\frac{1}{\Gamma (\mu )}\int _{0}^{\tau }(\tau -\varpi )^{\mu -1}\big [\mathcal {Z}_{1}(\chi ,\varpi ,\Phi _{\kappa -1}, \Psi _{\kappa -1})-\mathcal {Z}_{1}(\chi ,\varpi ,\Phi _{\kappa -2},\Psi _{\kappa -2})\big ]d\varpi ,\\ \Xi ^{2}_{\kappa }(\chi ,\tau ):=&\Psi _{\kappa }(\chi ,\tau )-\Psi _{\kappa -1}(\chi ,\tau )=\frac{1}{\Gamma (\mu )}\int _{0}^{\tau } (\tau -\varpi )^{\mu -1}\big [\mathcal {Z}_{2}(\chi ,\varpi ,\Phi _{\kappa -1},\Psi _{\kappa -1})-\mathcal {Z}_{2}(\chi ,\varpi ,\Phi _{\kappa -2},\Psi _{\kappa -2})\big ]d\varpi . \end{aligned}\right. \end{aligned}$$

Clearly,

34 $$\begin{aligned} \Phi _{\kappa }(\chi ,\tau )=\sum _{k=0}^{\kappa }\Xi ^{1}_{\kappa }(\chi ,\tau ), \ \ \ \Psi _{\kappa }(\chi ,\tau )=\sum _{k=0}^{\kappa }\Xi ^{2}_{\kappa }(\chi ,\tau ). \end{aligned}$$

By taking the norm in each of equation (33), the triangle inequality and the knowledge that $\mathcal {Z}_{1}$ and $\mathcal {Z}_{2}$ satisfy the LC result gives

35 $$\begin{aligned} \left\{ \begin{aligned}&\Vert \Xi ^{1}_{\kappa }(\chi ,\tau )\Vert \le \frac{1}{\Gamma (\mu )}\mathcal {L}_{1} \int _{0}^{\tau }(\tau -\varpi )^{\mu -1}\Vert \Xi ^{1}_{\kappa -1}(\chi ,\varpi )\Vert d\varpi ,\\&\Vert \Xi ^{2}_{\kappa }(\chi ,\tau )\Vert \le \frac{1}{\Gamma (\mu )} \mathcal {L}_{2}\int _{0}^{\tau }(\tau -\varpi )^{\mu -1}\Vert \Xi ^{2}_{\kappa -1}(\chi ,\varpi )\Vert d\varpi . \end{aligned}\right. \end{aligned}$$

Using the recursive approach and taking into account each of the inequalities in (35), we acquire

36 $$\begin{aligned} \left\{ \begin{aligned} \Vert \Xi ^{1}_{\kappa }(\chi ,\tau )\Vert \le&\left[ \frac{\tau ^{\mu }}{\Gamma (\mu +1)}\mathcal {L}_{1}\right] ^{\kappa }\Vert \Phi (\chi ,0)\Vert ,\\ \Vert \Xi ^{2}_{\kappa }(\chi ,\tau )\Vert \le&\left[ \frac{\tau ^{\mu }}{\Gamma (\mu +1)}\mathcal {L}_{2}\right] ^{\kappa }\Vert \Psi (\chi ,0)\Vert . \end{aligned}\right. \end{aligned}$$

This establishes the functions in (34), as well as their presence and smoothness. We define the following to demonstrate that these functions are solutions to (20):

37 $$\begin{aligned} \begin{aligned}&\Phi (\chi ,\tau )-\Phi (\chi ,0)=\Phi _{\kappa }(\chi ,\tau )-\mathcal {H}^{1}_{\kappa }(\chi ,\tau ),\\&\Psi (\chi ,\tau )-\Psi (\chi ,0)=\Psi _{\kappa }(\chi ,\tau )-\mathcal {H}^{2}_{\kappa }(\chi ,\tau ). \end{aligned} \end{aligned}$$

Then we acquire

$$\begin{aligned} \begin{aligned} \Vert \mathcal {H}^{1}_{\kappa }(\chi ,\tau )\Vert =&\left\| \frac{1}{\Gamma (\mu )}\int _{0}^{\tau } (\tau -\varpi )^{\mu -1}\big [\mathcal {Z}_{1}(\chi ,\varpi ,\Phi ,\Psi ) -\mathcal {Z}_{1}(\chi ,\varpi ,\Phi ,_{\kappa -1},\Psi _{\kappa -1})\big ]d\varpi \right\| \\ \le&\frac{1}{\Gamma (\mu )}\int _{0}^{\tau }(\tau -\varpi )^{\mu -1}\Vert \mathcal {Z}_{1}(\chi ,\varpi ,\Phi ,\Psi )-\mathcal {Z}_{1}(\chi ,\varpi ,\Phi ,_{\kappa -1},\Psi _{\kappa -1})\Vert d\varpi \\ \le&\frac{\tau ^{\mu }}{\Gamma (\mu +1)}\mathcal {L}_{1}\Vert \Phi -\Phi _{\kappa -1}\Vert . \end{aligned} \end{aligned}$$

Recursively repeating the procedure at $t=t _{0}$ results in

$$\begin{aligned} \begin{aligned} \Vert \mathcal {H}^{1}_{\kappa }(\chi ,\tau )\Vert \le&\left( \frac{\tau ^{\mu }_{0}}{\Gamma (\mu +1)}\right) ^{\kappa +1}\mathcal {L}_{1}^{\kappa +1}M. \end{aligned} \end{aligned}$$

Similarly, it is also demonstrable that

$$\begin{aligned} \begin{aligned} \Vert \mathcal {H}^{2}_{\kappa }(\chi ,\tau )\Vert \le&\left( \frac{\tau ^{\mu }_{0}}{\Gamma (\mu +1)}\right) ^{\kappa +1}\mathcal {L}_{2}^{\kappa +1}M. \end{aligned} \end{aligned}$$

As $\kappa \rightarrow \infty$, we have that $\Vert H^{i}_{\kappa }(\chi ,\tau )\Vert \rightarrow 0, \ i=1, 2$. This shows that there are solutions. To demonstrate the existence of a unique solution, let $\Phi _{1}(\chi ,\tau )$ and $\Psi _{1}(\chi ,\tau )$ be any two distinct solutions of (20). Next, we acquire

$$\begin{aligned} \begin{aligned} \Vert \Phi (\chi ,\tau )-\Phi _{1}(\chi ,\tau )\Vert \le&\frac{1}{\Gamma (\mu )}\int _{0}^{\tau } (\tau -\varpi )^{\mu -1}\Vert \mathcal {Z}_{1}(\chi ,\varpi ,\Phi ,\Psi )-\mathcal {Z}_{1}(\chi ,\varpi ,\Phi ,\Psi _{1})\Vert d\varpi \\ \le&\frac{\tau ^{\mu }}{\Gamma (\mu +1)}\mathcal {L}_{1}\Vert \Phi (\chi ,\tau )-\Phi _{1}(\chi ,\tau )\Vert . \end{aligned} \end{aligned}$$

Equivalently, we have

38 $$\begin{aligned} \begin{aligned} \Vert \Phi (\chi ,\tau )-\Phi _{1}(\chi ,\tau )\Vert \left( 1-\frac{\tau ^{\mu }}{\Gamma (\mu +1)}\mathcal {L}_{1}\right) \le 0. \end{aligned} \end{aligned}$$

Likewise, it may also be demonstrated that

39 $$\begin{aligned} \begin{aligned} \Vert \Psi (\chi ,\tau )-\Psi _{1}(\chi ,\tau )\Vert \left( 1-\frac{\tau ^{\mu }}{\Gamma (\mu +1)}\mathcal {L}_{2}\right) \le 0. \end{aligned} \end{aligned}$$

A similar line of argument leading to (29)–(39) also yield

40 $$\begin{aligned} \begin{aligned} \Vert \Phi (\chi ,\tau )-\Phi _{1}(\chi ,\tau )\Vert \left( 1-\frac{2(1-\mu )}{(2-\mu ) \mathcal {M}(\mu )}\mathcal {L}_{1}-\frac{2\mu }{(2-\mu )\mathcal {M}(\mu )}\mathcal {L}_{1}\tau \right) \le 0, \end{aligned} \end{aligned}$$

41 $$\begin{aligned} \begin{aligned} \Vert \Psi (\chi ,\tau )-\Psi _{1}(\chi ,\tau )\Vert \left( 1-\frac{2(1-\mu )}{(2-\mu ) \mathcal {M}(\mu )}\mathcal {L}_{2}-\frac{2\mu }{(2-\mu )\mathcal {M}(\mu )}\mathcal {L}_{2}\tau \right) \le 0, \end{aligned} \end{aligned}$$

if the TF-BZ reaction system (4) is considered in the $\texttt{CF}$ and

42 $$\begin{aligned} \begin{aligned} \Vert \Phi (\chi ,\tau )-\Phi _{1}(\chi ,\tau )\Vert \left( 1-\frac{1-\mu }{\mathcal {B}(\mu )}\mathcal {L}_{1}-\frac{\tau ^{\mu }}{\mathcal {B}(\mu )\Gamma (\mu )}\mathcal {L}_{1}\right) \le 0, \end{aligned} \end{aligned}$$

43 $$\begin{aligned} \begin{aligned} \Vert \Psi (\chi ,\tau )-\Psi _{1}(\chi ,\tau )\Vert \left( 1-\frac{1-\mu }{\mathcal {B}(\mu )} \mathcal {L}_{2}-\frac{\tau ^{\mu }}{\mathcal {B}(\mu )\Gamma (\mu )}\mathcal {L}_{2}\right) \le 0, \end{aligned} \end{aligned}$$

if it is considered in the $\texttt{AB}$ sense.

Therefore, the inequalities (38)–(43) suggest that $\Vert \Phi (\chi ,\tau )-\Phi _{1}(\chi ,\tau )\Vert =0$ and $\Vert \Psi (\chi ,\tau )-\Psi _{1}(\chi ,\tau )\Vert =0$ if and only if (28) is met. Hence, $\Phi (\chi ,\tau )=\Phi _{1}(\chi ,\tau )$ and $\Psi (\chi ,\tau )=\Psi _{1}(\chi ,\tau )$. Therefore (20) has a unique solution. Consequently, the TF-BZ reaction system possesses a unique solution. $\square$

## NTDM solution procedure

Given the following coupled system of time-fractional nonlinear PDEs

44 $$\begin{aligned} \begin{aligned}&^{\Xi (\mu )}\textrm{D}_{\tau }^{\mu }\Phi (\chi ,\tau )+\Im _{1}[\Phi (\chi ,\tau ), \Psi (\chi ,\tau )]+\aleph _{1}[\Phi (\chi ,\tau ),\Psi (\chi ,\tau )]=\mathbb {G}(\chi ,\tau ),\\&^{\Xi (\mu )}\textrm{D}_{\tau }^{\mu }\Psi (\chi ,\tau )+\Im _{2}[\Phi (\chi ,\tau ), \Psi (\chi ,\tau )]+\aleph _{2}[\Phi (\chi ,\tau ),\Psi (\chi ,\tau )]=\mathbb {H}(\chi ,\tau ),\\&\Phi (\chi ,0)=\Phi _{0}(\chi ),\ \Psi (\chi ,0)=\Psi _{0}(\chi ), \end{aligned} \end{aligned}$$

where $$0<\mu \le 1$$, $$^{\Xi (\mu )}\textrm{D}^{\mu }_{\tau }$$ denotes either the Caputo, $$\texttt{CF}$$ or $$\texttt{AB}$$ derivative, $$\Im _{i}$$ and $$\aleph _{i}$$$$(i=1, 2)$$ denote linear and nonlinear partial derivatives, respectively, while $$\mathbb {G}(\chi ,\tau )$$ and $$\mathbb {H}(\chi ,\tau )$$ signify non-homogeneous functions. By operating both sides of each equation in (44) with $$\mathbb{N}\mathbb{T}^{+}$$ we get

45 $$\begin{aligned} \begin{aligned}&\mathbb{N}\mathbb{T}^{+}\left[ ^{\Xi (\mu )}\textrm{D}_{\tau }^{\mu }\Phi (\chi ,\tau )\right] = -\mathbb{N}\mathbb{T}^{+}[\Im _{1}[\Phi (\chi ,\tau ),\Psi (\chi ,\tau )]]-\mathbb{N}\mathbb{T}^{+} [\aleph _{1}[\Phi (\chi ,\tau ),\Psi (\chi ,\tau )]]+\mathbb{N}\mathbb{T}^{+}[\mathbb {G}(\chi ,\tau )],\\&\mathbb{N}\mathbb{T}^{+}\left[ ^{\Xi (\mu )}\textrm{D}_{\tau }^{\mu }\Psi (\chi ,\tau )\right] = -\mathbb{N}\mathbb{T}^{+}[\Im _{2}[\Phi (\chi ,\tau ),\Psi (\chi ,\tau )]]- \mathbb{N}\mathbb{T}^{+}[\aleph _{2}[\Phi (\chi ,\tau ),\Psi (\chi ,\tau )]]+\mathbb{N}\mathbb{T}^{+}[\mathbb {H}(\chi ,\tau )]. \end{aligned} \end{aligned}$$

Thanks to the natural transform property (16)–(18) for the Caputo, $\texttt{CF}$ and $\texttt{AB}$ derivatives, (45) implies

46 $$\begin{aligned} \begin{aligned}&\frac{1}{{\textbf {F}}(\mu ,\omega ,s)}\left( \mathbb{N}\mathbb{T}^{+}[\Phi (\chi ,\tau )] -\frac{\Phi _{0}(\chi )}{s}\right) =\mathbb{N}\mathbb{T}^{+}\Big [-\Im _{1}[\Phi (\chi ,\tau ), \Psi (\chi ,\tau )]-\aleph _{1}[\Phi (\chi ,\tau ),\Psi (\chi ,\tau )]+\mathbb {G}(\chi ,\tau )\Big ],\\&\frac{1}{{\textbf {F}}(\mu ,\omega ,s)}\left( \mathbb{N}\mathbb{T}^{+}[\Psi (\chi ,\tau )] -\frac{\Psi _{0}(\chi )}{s}\right) =\mathbb{N}\mathbb{T}^{+}\Big [-\Im _{2}[\Phi (\chi ,\tau ), \Psi (\chi ,\tau )]-\aleph _{2}[\Phi (\chi ,\tau ),\Psi (\chi ,\tau )]+\mathbb {H}(\chi ,\tau )\Big ], \end{aligned} \end{aligned}$$

where

47 $$\begin{aligned} {\textbf {F}}(\mu ,\omega ,s)= \left\{ \begin{aligned}&\left( \frac{\omega }{s}\right) ^{\mu }, \ & \text{ if } \ \ \Xi (\mu )=\texttt{C},\\&1-\mu +\mu \left( \frac{\omega }{s}\right) , \ & \text{ if } \ \ \Xi (\mu )=\texttt{CF},\\&\frac{1-\mu +\mu \left( \frac{\omega }{s}\right) ^{\mu }}{\mathcal {B}(\mu )}, \ & \text{ if } \ \ \Xi (\mu )=\texttt{AB}. \end{aligned}\right. \end{aligned}$$

Operating the equations in (46) with $\mathbb{N}\mathbb{T}^{-1}$ yields

48 $$\begin{aligned} \begin{aligned}&\Phi (\chi ,\tau )=\mathbb{N}\mathbb{T}^{-1}\left[ \frac{\Phi _{0}(\chi )}{s}+{\textbf {F}}(\mu ,\omega ,s) \mathbb{N}\mathbb{T}^{+}\Big [-\Im _{1}[\Phi (\chi ,\tau ),\Psi (\chi ,\tau )]-\aleph _{1}[\Phi (\chi ,\tau ), \Psi (\chi ,\tau )]+\mathbb {G}(\chi ,\tau )\Big ]\right] ,\\&\Psi (\chi ,\tau )=\mathbb{N}\mathbb{T}^{-1}\left[ \frac{\Psi _{0}(\chi )}{s}+{\textbf {F}}(\mu ,\omega ,s) \mathbb{N}\mathbb{T}^{+}\Big [-\Im _{2}[\Phi (\chi ,\tau ),\Psi (\chi ,\tau )]-\aleph _{2}[\Phi (\chi ,\tau ),\Psi (\chi ,\tau )] +\mathbb {H}(\chi ,\tau )\Big ]\right] . \end{aligned} \end{aligned}$$

The NTDM assumes a solution of the system of equations (44) in the form of the infinite series

49 $$\begin{aligned} \Phi (\chi ,\tau )=\sum _{\kappa =0}^{\infty }\Phi _{\kappa }(\chi ,\tau ), \ \ \Psi (\chi ,\tau )=\sum _{\kappa =0}^{\infty }\Psi _{\kappa }(\chi ,\tau ), \end{aligned}$$

while the nonlinear terms are given by the decomposition series

50 $$\begin{aligned} \aleph _{1}[\Phi (\chi ,\tau ),\Psi (\chi ,\tau )]=\sum _{\kappa =0}^{\infty } {\textbf {A}}_{\kappa }(\chi ,\tau ), \ \ \aleph _{2}[\Phi (\chi ,\tau ),\Psi (\chi ,\tau )]=\sum _{\kappa =0}^{\infty } {\textbf {B}}_{\kappa }(\chi ,\tau ), \end{aligned}$$

where ${\textbf {A}}_{\kappa }$ and ${\textbf {B}}_{\kappa }$ are Adomian polynomials defined by

$${\textbf {A}}_{\kappa }=\frac{1}{\Gamma (\kappa +1)}\left[ \frac{d^{\kappa }}{d\lambda ^{\kappa }}\left[ \aleph _{1}\left( \sum _{\kappa =0}^{\infty } \lambda ^{\kappa }\Phi _{\kappa }(\chi ,\tau ),\sum _{\kappa =0}^{\infty }\lambda ^{\kappa }\Psi _{\kappa }(\chi ,\tau )\right) \right] \right] _{\lambda =0},$$

and

$${\textbf {B}}_{\kappa }=\frac{1}{\Gamma (\kappa +1)}\left[ \frac{d^{\kappa }}{d\lambda ^{\kappa }}\left[ \aleph _{2}\left( \sum _{\kappa =0}^{\infty } \lambda ^{\kappa }\Phi _{\kappa }(\chi ,\tau ),\sum _{\kappa =0}^{\infty }\lambda ^{\kappa }\Psi _{\kappa }(\chi ,\tau )\right) \right] \right] _{\lambda =0},$$

respectively. Inserting (49) and (50) into (48) gives

51 $$\begin{aligned} \begin{aligned} \sum _{\kappa =0}^{\infty }\Phi _{\kappa }(\chi ,\tau )=&\mathbb{N}\mathbb{T}^{-1}\left[ \frac{\Phi _{0}(\chi )}{s} +{\textbf {F}}(\mu ,\omega ,s)\mathbb{N}\mathbb{T}^{+}[\mathbb {G}(\chi ,\tau )]\right] \\&-\mathbb{N}\mathbb{T}^{-1}\left[ {\textbf {F}}(\mu ,\omega ,s)\mathbb{N}\mathbb{T}^{+}\left[ \Im _{1} \left[ \sum _{\kappa =0}^{\infty }\Phi _{\kappa }(\chi ,\tau ),\sum _{\kappa =0}^{\infty } \Psi _{\kappa }(\chi ,\tau )\right] +\sum _{\kappa =0}^{\infty } {\textbf {A}}_{\kappa }\right] \right] ,\\ \sum _{\kappa =0}^{\infty }\Psi _{\kappa }(\chi ,\tau )=&\mathbb{N}\mathbb{T}^{-1}\left[ \frac{\Psi _{0}(\chi )}{s} +{\textbf {F}}(\mu ,\omega ,s)\mathbb{N}\mathbb{T}^{+}[\mathbb {H}(\chi ,\tau )]\right] \\&-\mathbb{N}\mathbb{T}^{-1}\left[ {\textbf {F}}(\mu ,\omega ,s)\mathbb{N}\mathbb{T}^{+}\left[ \Im _{2} \left[ \sum _{\kappa =0}^{\infty }\Phi _{\kappa }(\chi ,\tau ),\sum _{\kappa =0}^{\infty }\Psi _{\kappa }(\chi ,\tau )\right] +\sum _{\kappa =0}^{\infty } {\textbf {B}}_{\kappa }\right] \right] . \end{aligned} \end{aligned}$$

Equating terms on both sides of each equation in (51) yields the recurrence relation

52 $$\begin{aligned} \begin{aligned}&\Phi ^{{\Xi (\mu )}}_{0}(\chi ,\tau )=\mathbb{N}\mathbb{T}^{-1}\left[ \frac{\Phi _{0}(\chi )}{s} +{\textbf {F}}(\mu ,\omega ,s)\mathbb{N}\mathbb{T}^{+}[\mathbb {G}(\chi ,\tau )]\right] ,\\&\Psi ^{{\Xi (\mu )}}_{0}(\chi ,\tau )=\mathbb{N}\mathbb{T}^{-1}\left[ \frac{\Psi _{0}(\chi )}{s} +{\textbf {F}}(\mu ,\omega ,s)\mathbb{N}\mathbb{T}^{+}[\mathbb {H}(\chi ,\tau )]\right] ,\\&\Phi ^{{\Xi (\mu )}}_{\kappa +1}(\chi ,\tau )=-\mathbb{N}\mathbb{T}^{-1}\left[ {\textbf {F}}(\mu ,\omega ,s)\mathbb{N}\mathbb{T}^{+}\left[ \Im _{1}[\Phi _{\kappa }(\chi ,\tau ),\Psi _{\kappa }(\chi ,\tau )]+{\textbf {A}}_{\kappa }\right] \right] ,\ \kappa \ge 1,\\&\Psi ^{{\Xi (\mu )}}_{\kappa +1}(\chi ,\tau )=-\mathbb{N}\mathbb{T}^{-1}\left[ {\textbf {F}}(\mu ,\omega ,s)\mathbb{N}\mathbb{T}^{+}\left[ \Im _{2}[\Phi _{\kappa }(\chi ,\tau ),\Psi _{\kappa }(\chi ,\tau )]+{\textbf {B}}_{\kappa }\right] \right] ,\ \kappa \ge 1. \end{aligned} \end{aligned}$$

After substituting (52) into (49), the approximate solution for the coupled system (44) is given as

53 $$\begin{aligned} \begin{aligned}&\Phi ^{{\Xi (\mu )}}(\chi ,\tau )=\sum _{\kappa =0}^{\infty }\Phi ^{{\Xi (\mu )}}_{\kappa }(\chi ,\tau )=\Phi ^{{\Xi (\mu )}}_{0}(\chi ,\tau )+\Phi ^{{\Xi (\mu )}}_{1}(\chi ,\tau )+\Phi ^{{\Xi (\mu )}}_{2}(\chi ,\tau )+\Phi ^{{\Xi (\mu )}}_{3}(\chi ,\tau )+\cdots ,\\&\Psi ^{{\Xi (\mu )}}(\chi ,\tau )=\sum _{\kappa =0}^{\infty }\Psi ^{{\Xi (\mu )}}_{\kappa }(\chi ,\tau )=\Psi ^{{\Xi (\mu )}}_{0}(\chi ,\tau )+\Psi ^{{\Xi (\mu )}}_{1}(\chi ,\tau )+\Psi ^{{\Xi (\mu )}}_{2}(\chi ,\tau )+\Psi ^{{\Xi (\mu )}}_{3}(\chi ,\tau )+\cdots . \end{aligned} \end{aligned}$$

### Remark 1

We refer to^50^ for results concerning the convergence analysis of the NTDM for the Caputo, $\texttt{CF}$ and $\texttt{AB}$ derivatives.

## Numerical implementation and results

In this section, two versions of the TF-BZ reaction system (4) are studied within the framework of Caputo, $\texttt{CF}$ and $\texttt{AB}$ derivatives using in considered method.

### Problem 1

Suppose $\varrho _{1}=\varrho _{2}=1$ and $\gamma =\beta =0$, $\zeta \ne 1$, we consider the following version of the TF-BZ reaction system subject to

54 $$\begin{aligned} \left\{ \begin{aligned}&^{{\Xi (\mu )}}\textrm{D}_{\tau }^{\mu }\Phi -\Phi _{\chi \chi }-\Phi +\Phi ^{2}+\xi \Phi \Psi =0,\\&^{{\Xi (\mu )}}\textrm{D}_{\tau }^{\mu }\Psi -\Psi _{\chi \chi }+\zeta \Phi \Psi =0,\\&\Phi (\chi ,0)=\frac{1}{\left( e^{\sqrt{\frac{\zeta }{6}}\chi }+1\right) ^{2}}, \ \ \Psi (\chi ,0)=\frac{(1-\zeta )e^{\sqrt{\frac{\zeta }{6}}\chi }\left( e^{\sqrt{\frac{\zeta }{6}}\chi }+2\right) }{\xi \left( e^{\sqrt{\frac{\zeta }{6}}\chi }+1\right) ^{2}}. \end{aligned}\right. \end{aligned}$$

Here, ${\Xi (\mu )}$ represents either the Caputo, $\texttt{CF}$ or $\texttt{AB}$ derivative. When $\mu =1$, the exact solution for (54) is

55 $$\begin{aligned} \Phi (\chi ,\tau )=\frac{e^{\frac{5\zeta }{3}\tau }}{\left( e^{\sqrt{\frac{\zeta }{6}}\chi }+e^{\frac{5\zeta }{6}\tau }\right) ^{2}}, \ \ \Psi (\chi ,\tau )=\frac{(1-\zeta )e^{\sqrt{\frac{\zeta }{6}}\chi }\left( e^{\sqrt{\frac{\zeta }{6}}\chi }+2e^{\frac{5\zeta }{6}\tau }\right) }{\xi \left( e^{\sqrt{\frac{\zeta }{6}}\chi }+e^{\frac{5\zeta }{6}\tau }\right) ^{2}}. \end{aligned}$$

Next, by defining the solutions $\Phi (\chi ,\tau )$ and $\Psi (\chi ,\tau )$ in form of the infinite series

$$\Phi (\chi ,\tau )=\sum _{\kappa =0}^{\infty }\Phi _{\kappa }, \ \ \Psi =\sum _{\kappa =0}^{\infty }\Psi _{\kappa },$$

and representing the nonlinear terms $\Phi ^{2}$ and $\Phi \Psi$ by the Adomian polynomials

$$\Phi ^{2}=\sum _{\kappa =0}^{\infty } {\textbf {A}}_{\kappa }, \ \ \Phi \Psi =\sum _{\kappa =0}^{\infty } {\textbf {B}}_{\kappa },$$

with some of its components as

56 $$\begin{aligned} \begin{aligned}&{\textbf {A}}_{0}=\Phi _{0}^{2},\\&{\textbf {A}}_{1}=2\Phi _{0}\Phi _{1},\\&{\textbf {A}}_{2}=2\Phi _{0}\Phi _{2}+\Phi _{1}^{2},\\&{\textbf {A}}_{3}=2\Phi _{0}\Phi _{3}+2\Phi _{1}\Phi _{2}, \end{aligned} \quad \quad \quad \quad \begin{aligned}&{\textbf {B}}_{0}=\Phi _{0}\Psi _{0},\\&{\textbf {B}}_{1}=\Phi _{0}\Psi _{1}+\Phi _{1}\Psi _{0},\\&{\textbf {B}}_{2}=\Phi _{0}\Psi _{2}+\Phi _{1}\Psi _{1}+\Phi _{2}\Psi _{1},\\&{\textbf {B}}_{3}=\Phi _{0}\Psi _{3}+\Phi _{1}\Psi _{2}+\Phi _{2}\Psi _{1}+\Phi _{3}\Psi _{0}, \end{aligned} \end{aligned}$$

the NTDM procedure leading to (51) yields

57 $$\begin{aligned} \begin{aligned} \sum _{\kappa =0}^{\infty }\Phi _{\kappa }^{{\Xi (\mu )}}(\chi ,\tau )=&\frac{1}{\left( e^{\sqrt{\frac{\zeta }{6}}\chi }+1\right) ^{2}} -\mathbb{N}\mathbb{T}^{-1}\left[ {\textbf {F}}(\mu ,\omega ,s)\mathbb{N}\mathbb{T}^{+}\left[ -\sum _{\kappa =0}^{\infty }\frac{\partial ^{2}\Phi _{\kappa }(\chi ,\tau )}{\partial \chi ^{2}}-\Phi _{\kappa }(\chi ,\tau )+\sum _{\kappa =0}^{\infty } {\textbf {A}}_{\kappa }(\chi ,\tau )+\xi \sum _{\kappa =0}^{\infty } {\textbf {B}}_{\kappa }(\chi ,\tau )\right] \right] ,\\ \sum _{\kappa =0}^{\infty }\Psi _{\kappa }^{{\Xi (\mu )}}(\chi ,\tau )=&\frac{(1-\zeta )e^{\sqrt{\frac{\zeta }{6}}\chi }\left( e^{\sqrt{\frac{\zeta }{6}}\chi }+2\right) }{\xi \left( e^{\sqrt{\frac{\zeta }{6}}\chi }+1\right) ^{2}} -\mathbb{N}\mathbb{T}^{-1}\left[ {\textbf {F}}(\mu ,\omega ,s)\mathbb{N}\mathbb{T}^{+}\left[ -\sum _{\kappa =0}^{\infty }\frac{\partial ^{2}\Psi _{\kappa }(\chi ,\tau )}{\partial \chi ^{2}}+\zeta \sum _{\kappa =0}^{\infty } {\textbf {B}}_{\kappa }(\chi ,\tau )\right] \right] , \end{aligned} \end{aligned}$$

for the considered problem (54). Furthermore, we obtain the following recursive equations from (57):

58 $$\begin{aligned} \begin{aligned}&\Phi _{0}^{{\Xi (\mu )}}(\chi ,\tau )=\frac{1}{\left( e^{\sqrt{\frac{\zeta }{6}}\chi }+1\right) ^{2}},\\&\Psi _{0}^{{\Xi (\mu )}}(\chi ,\tau )=\frac{(1-\zeta )e^{\sqrt{\frac{\zeta }{6}}\chi }\left( e^{\sqrt{\frac{\zeta }{6}}\chi }+2\right) }{\xi \left( e^{\sqrt{\frac{\zeta }{6}}\chi }+1\right) ^{2}},\\&\Phi _{\kappa +1}^{{\Xi (\mu )}}(\chi ,\tau )=-\mathbb{N}\mathbb{T}^{-1}\left[ {\textbf {F}}(\mu ,\omega ,s)\mathbb{N}\mathbb{T}^{+}\left[ -\frac{\partial ^{2}\Phi _{\kappa }(\chi ,\tau )}{\partial \chi ^{2}}-\Phi _{\kappa }(\chi ,\tau )+{\textbf {A}}_{\kappa }(\chi ,\tau )+\xi {\textbf {B}}_{\kappa }(\chi ,\tau )\right] \right] ,\\&\Psi _{\kappa +1}^{{\Xi (\mu )}}(\chi ,\tau )=-\mathbb{N}\mathbb{T}^{-1}\left[ {\textbf {F}}(\mu ,\omega ,s)\mathbb{N}\mathbb{T}^{+}\left[ -\frac{\partial ^{2}\Psi _{\kappa }(\chi ,\tau )}{\partial \chi ^{2}}+\zeta {\textbf {B}}_{\kappa }(\chi ,\tau )\right] \right] , \ \ \ \kappa \ge 1. \end{aligned} \end{aligned}$$

#### NTDM series solution for (54) in Caputo sense:

By setting

$${\textbf {F}}(\mu ,\omega ,s)=\left( \frac{\omega }{s}\right) ^{\mu },$$

in (58), some few iterations obtained are

$$\begin{aligned} \begin{aligned} \Phi ^{\texttt{C}}_{0}(\chi ,\tau )=&\frac{1}{\left( e^{\sqrt{\frac{\zeta }{6}}\chi }+1\right) ^{2}}, \qquad & \Psi ^{\texttt{C}}_{0}(\chi ,\tau )=\frac{(1-\zeta )e^{\sqrt{\frac{\zeta }{6}}\chi } \left( e^{\sqrt{\frac{\zeta }{6}}\chi }+2\right) }{\xi \left( e^{\sqrt{\frac{\zeta }{6}}\chi }+1\right) ^{2}},\\ \Phi ^{\texttt{C}}_{1}(\chi ,\tau )=&\frac{5 \zeta \tau ^{\mu } e^{\sqrt{\frac{\zeta }{6}}\chi }}{3 \Gamma (\mu +1) \left( e^{\sqrt{\frac{\zeta }{6}}\chi }+1\right) ^3},\qquad & \Psi ^{\texttt{C}}_{1}(\chi ,\tau )=\frac{5 (\zeta -1) \zeta \tau ^{\mu } e^{\sqrt{\frac{\zeta }{6}}\chi }}{3 \xi \Gamma (\mu +1) \left( e^{\sqrt{\frac{\zeta }{6}}\chi }+1\right) ^3},\\ \Phi ^{\texttt{C}}_{2}(\chi ,\tau )=&\frac{25 \zeta ^2 \tau ^{2\mu } e^{\sqrt{\frac{\zeta }{6}}\chi } \left( 2 e^{\sqrt{\frac{\zeta }{6}}\chi }-1\right) }{18 \Gamma (2 \mu +1) \left( e^{\sqrt{\frac{\zeta }{6}}\chi }+1\right) ^4},\qquad & \Psi ^{\texttt{C}}_{2}(\chi ,\tau )=\frac{25 (\zeta -1) \zeta ^2 \tau ^{2\mu } e^{\sqrt{\frac{\zeta }{6}}\chi } \left( 2 e^{\sqrt{\frac{\zeta }{6}}\chi }-1\right) }{18 \xi \Gamma (2 \mu +1) \left( e^{\sqrt{\frac{\zeta }{6}}\chi }+1\right) ^4}. \end{aligned} \end{aligned}$$

Similar expressions for $\Phi ^{\texttt{C}}_{\kappa }(\chi ,\tau )$ and $\Psi ^{\texttt{C}}_{\kappa }(\chi ,\tau )$ for $\kappa \ge 3$ can also be obtained using the recurrence relation in (58). Furthermore, we have

59 $$\begin{aligned} \begin{aligned} \Phi ^{\texttt{C}}(\chi ,\tau )=&\Phi ^{\texttt{C}}_{0}(\chi ,\tau )+\Phi ^{\texttt{C}}_{1}(\chi ,\tau ) +\Phi ^{\texttt{C}}_{2}(\chi ,\tau )+\Phi ^{\texttt{C}}_{3}(\chi ,\tau )+\cdots ,\\ \Psi ^{\texttt{C}}(\chi ,\tau )=&\Psi ^{\texttt{C}}_{0}(\chi ,\tau )+\Psi ^{\texttt{C}}_{1}(\chi ,\tau ) +\Psi ^{\texttt{C}}_{2}(\chi ,\tau )+\Psi ^{\texttt{C}}_{3}(\chi ,\tau )+\cdots , \end{aligned} \end{aligned}$$

as the solution obtained by the NTDM for fractional system (54) in the Caputo sense.

#### NTDM series solution for (54) in $\texttt{CF}$ sense:

By setting

$${\textbf {F}}(\mu ,\omega ,s)=1-\mu +\mu \left( \frac{\omega }{s}\right) ,$$

in (58), we generate the following few iterations

$$\begin{aligned} \begin{aligned} \Phi ^{\texttt{CF}}_{0}(\chi ,\tau )=&\frac{1}{\xi \left( e^{\sqrt{\frac{\zeta }{6}}\chi }+1\right) ^{2}},\qquad \qquad \qquad \quad \Psi ^{\texttt{CF}}_{0}(\chi ,\tau )=\frac{(1-\zeta )e^{\sqrt{\frac{\zeta }{6}}\chi }\left( e^{\sqrt{\frac{\zeta }{6}}\chi }+2\right) }{\xi \left( e^{\sqrt{\frac{\zeta }{6}}\chi }+1\right) ^{2}},\\ \Phi ^{\texttt{CF}}_{1}(\chi ,\tau )=&\frac{5 \zeta (\mu (\tau -1)+1) e^{\sqrt{\frac{\zeta }{6}}\chi }}{3 \left( e^{\sqrt{\frac{\zeta }{6}}\chi }+1\right) ^3},\qquad \qquad \Psi ^{\texttt{CF}}_{1}(\chi ,\tau )=\frac{5 (\zeta -1) \zeta (\mu (\tau -1)+1) e^{\sqrt{\frac{\zeta }{6}}\chi }}{3 \xi \left( e^{\sqrt{\frac{\zeta }{6}}\chi }+1\right) ^3},\\ \Phi ^{\texttt{CF}}_{2}(\chi ,\tau )=&\frac{25 \zeta ^2 \left( \mu ^2 \left( \tau ^2-4 \tau +2\right) +4\mu (\tau -1)+2\right) e^{\sqrt{\frac{\zeta }{6}}\chi } \left( 2 e^{\sqrt{\frac{\zeta }{6}}\chi }-1\right) }{36 \left( e^{\sqrt{\frac{\zeta }{6}}\chi }+1\right) ^4},\\ \Psi ^{\texttt{CF}}_{2}(\chi ,\tau )=&\frac{25 (\zeta -1) \zeta ^2 \left( \mu ^2 \left( \tau ^2-4 \tau +2\right) +4\mu (\tau -1)+2\right) e^{\sqrt{\frac{\zeta }{6}}\chi } \left( 2 e^{\sqrt{\frac{\zeta }{6}}\chi }-1\right) }{36 \xi \left( e^{\sqrt{\frac{\zeta }{6}}\chi }+1\right) ^4}. \end{aligned} \end{aligned}$$

Similar expressions for $\Phi ^{\texttt{CF}}_{\kappa }(\chi ,\tau )$ and $\Psi ^{\texttt{CF}}_{\kappa }(\chi ,\tau )$ for $\kappa \ge 3$ can also be obtained using (58). Furthermore, the NTDM series solution for (54) with $\texttt{CF}$ derivative is given according to

60 $$\begin{aligned} \begin{aligned} \Phi ^{\texttt{CF}}(\chi ,\tau )=&\Phi ^{\texttt{CF}}_{0}(\chi ,\tau ) +\Phi ^{\texttt{CF}}_{1}(\chi ,\tau )+\Phi ^{\texttt{CF}}_{2}(\chi ,\tau )+\Phi ^{\texttt{CF}}_{3}(\chi ,\tau )+\cdots ,\\ \Psi ^{\texttt{CF}}(\chi ,\tau )=&\Psi ^{\texttt{CF}}_{0}(\chi ,\tau ) +\Psi ^{\texttt{CF}}_{1}(\chi ,\tau )+\Psi ^{\texttt{CF}}_{2}(\chi ,\tau )+\Psi ^{\texttt{CF}}_{3}(\chi ,\tau )+\cdots . \end{aligned} \end{aligned}$$

#### NTDM series solution for (54) in $\texttt{AB}$ sense:

By setting

$${\textbf {F}}(\mu ,\omega ,s)=\frac{1-\mu +\mu \left( \frac{\omega }{s}\right) ^{\mu }}{\mathcal {B}(\mu )},$$

in (58) with $\mathcal {B}(\mu )=1$ we generate the following iterates

$$\begin{aligned} \begin{aligned} \Phi ^{\texttt{AB}}_{0}(\chi ,\tau )=&\frac{1}{\xi \left( e^{\sqrt{\frac{\zeta }{6}}\chi }+1\right) ^{2}},\qquad \qquad \qquad \qquad \quad \ \Psi ^{\texttt{AB}}_{0}(\chi ,\tau )=\frac{(1-\zeta )e^{\sqrt{\frac{\zeta }{6}}\chi }\left( e^{\sqrt{\frac{\zeta }{6}}\chi }+2\right) }{\xi \left( e^{\sqrt{\frac{\zeta }{6}}\chi }+1\right) ^{2}},\\ \Phi _1^{\texttt{AB}}(\chi ,\tau )=&\frac{5 \zeta e^{\sqrt{\frac{\zeta }{6}}\chi } \left( \mu \tau ^{\mu }-(\mu -1) \Gamma (\mu +1)\right) }{3 \Gamma (\mu +1) \left( e^{\sqrt{\frac{\zeta }{6}}\chi }+1\right) ^3},\qquad \Psi _1^{\texttt{AB}}(\chi ,\tau )=\frac{5 (\zeta -1) \zeta e^{\sqrt{\frac{\zeta }{6}}\chi } \left( \mu \tau ^{\mu }-(\mu -1) \Gamma (\mu +1)\right) }{3 \xi \Gamma (\mu +1) \left( e^{\sqrt{\frac{\zeta }{6}}\chi }+1\right) ^3},\\ \Phi _2^{\texttt{AB}}(\chi ,\tau )=&\frac{25 \zeta ^2 e^{\sqrt{\frac{\zeta }{6}}\chi } \left( 2 e^{\sqrt{\frac{\zeta }{6}}\chi }-1\right) \left( \Gamma (\mu +1) \left( (\mu -1)^2 \Gamma (2 \mu +1)+\mu ^2 \tau ^{2\mu }\right) -2 (\mu -1) \mu \Gamma (2 \mu +1) \tau ^{\mu }\right) }{18 \Gamma (\mu +1) \Gamma (2 \mu +1) \left( e^{\sqrt{\frac{\zeta }{6}}\chi }+1\right) ^4},\\ \Psi _2^{\texttt{AB}}(\chi ,\tau )=&\frac{25 (\zeta -1) \zeta ^2 e^{\sqrt{\frac{\zeta }{6}}\chi } \left( 2 e^{\sqrt{\frac{\zeta }{6}}\chi }-1\right) \left( \Gamma (\mu +1) \left( (\mu -1)^2 \Gamma (2 \mu +1)+\mu ^2 \tau ^{2\mu }\right) -2 (\mu -1) \mu \Gamma (2 \mu +1) \tau ^{\mu }\right) }{18 \xi \Gamma (\mu +1) \Gamma (2 \mu +1) \left( e^{\sqrt{\frac{\zeta }{6}}\chi }+1\right) ^4}. \end{aligned} \end{aligned}$$

Similar expressions for $\Phi ^{\texttt{AB}}_{\kappa }(\chi ,\tau )$ and $\Psi ^{\texttt{AB}}_{\kappa }(\chi ,\tau )$ for $\kappa \ge 3$ can also be obtained using (58). Furthermore, the NTDM series solution for (54) with $\texttt{AB}$ derivative is given according to

61 $$\begin{aligned} \begin{aligned} \Phi ^{\texttt{AB}}(\chi ,\tau )=&\Phi ^{\texttt{AB}}_{0}(\chi ,\tau )+\Phi ^{\texttt{AB}}_{1}(\chi ,\tau )+\Phi ^{\texttt{AB}}_{2}(\chi ,\tau )+\Phi ^{\texttt{AB}}_{3}(\chi ,\tau )+\cdots ,\\ \Psi ^{\texttt{AB}}(\chi ,\tau )=&\Psi ^{\texttt{AB}}_{0}(\chi ,\tau )+\Psi ^{\texttt{AB}}_{1}(\chi ,\tau )+\Psi ^{\texttt{AB}}_{2}(\chi ,\tau )+\Psi ^{\texttt{AB}}_{3}(\chi ,\tau )+\cdots . \end{aligned} \end{aligned}$$

### Problem 2

Let $\varrho _{1}=\varrho _{2}=1$, $\gamma =\zeta$ and $\beta =1$, then we consider the following version of the TF-BZ reaction system with the given initial conditions

62 $$\begin{aligned} \left\{ \begin{aligned}&^{{\Xi (\mu )}}\textrm{D}_{\tau }^{\mu }\Phi =\Phi _{\chi \chi }+\xi \Psi +\Phi -\Phi ^{2}-\xi \Phi \Psi , \\&^{{\Xi (\mu )}}\textrm{D}_{\tau }^{\mu }\Psi =\Psi _{\chi \chi }+\zeta \Psi -\zeta \Phi \Psi ,\\&\Phi (\chi ,0)=\frac{1}{\left( e^{\sqrt{\frac{\zeta }{6}}\chi }+1\right) ^{2}}, \ \ \Psi (\chi ,0)=\frac{\zeta -1}{\xi \left( e^{\sqrt{\frac{\zeta }{6}}\chi }+1\right) ^{2}}. \end{aligned}\right. \end{aligned}$$

Here, ${\Xi (\mu )}$ represents either the Caputo, $\texttt{CF}$ or $\texttt{AB}$ derivative. When $\mu =1$, the exact solution for (62) is

63 $$\begin{aligned} \Phi (\chi ,\tau )=\frac{e^{\frac{5\zeta }{3}\tau }}{\left( e^{\sqrt{\frac{\zeta }{6}}\chi }+e^{\frac{5\zeta }{6}\tau }\right) ^{2}}, \Psi (\chi ,\tau )=\frac{(\zeta -1)e^{\frac{5\zeta }{3}\tau }}{\xi \left( e^{\sqrt{\frac{\zeta }{6}}\chi }+e^{\frac{5\zeta }{6}\tau }\right) ^{2}}. \end{aligned}$$

Next, by defining the series

$$\Phi =\sum _{\kappa =0}^{\infty }\Phi _{\kappa }, \ \ \Psi =\sum _{\kappa =0}^{\infty }\Psi _{\kappa },$$

and representing the nonlinear terms $\Phi ^{2}$ and $\Phi \Psi$ by the Adomian polynomials

$$\Phi ^{2}=\sum _{\kappa =0}^{\infty } {\textbf {A}}_{\kappa }, \ \ \Phi \Psi =\sum _{\kappa =0}^{\infty } {\textbf {B}}_{\kappa },$$

the NTDM procedure leading to (51) yields

64 $$\begin{aligned} \begin{aligned} \sum _{\kappa =0}^{\infty }\Phi _{\kappa }^{{\Xi (\mu )}}(\chi ,\tau )=&\frac{1}{\left( e^{\sqrt{\frac{\zeta }{6}}\chi } +1\right) ^{2}}-\mathbb{N}\mathbb{T}^{-1}\left[ {\textbf {F}}(\mu ,\omega ,s)\mathbb{N}\mathbb{T}^{+} \left( -\sum _{\kappa =0}^{\infty }\frac{\partial ^{2}\Phi _{\kappa }(\chi ,\tau )}{\partial \chi ^{2}}\right. \right. \\&\left. \left. -\xi \sum _{\kappa =0}^{\infty }\Psi _{\kappa }(\chi ,\tau )-\sum _{\kappa =0}^{\infty } \Phi _{\kappa }(\chi ,\tau )+\sum _{\kappa =0}^{\infty } {\textbf {A}}_{\kappa }(\chi ,\tau )+\xi \sum _{\kappa =0}^{\infty } {\textbf {B}}_{\kappa }(\chi ,\tau )\right) \right] ,\\ \sum _{\kappa =0}^{\infty }\Psi _{\kappa }^{{\Xi (\mu )}}(\chi ,\tau )=&\frac{(1-\zeta ) e^{\sqrt{\frac{\zeta }{6}}\chi }\left( e^{\sqrt{\frac{\zeta }{6}}\chi }+2\right) }{\xi \left( e^{\sqrt{\frac{\zeta }{6}}\chi }+1\right) ^{2}}\\&-\mathbb{N}\mathbb{T}^{-1}\left[ {\textbf {F}}(\mu ,\omega ,s)\mathbb{N}\mathbb{T}^{+}\left( -\sum _{\kappa =0}^{\infty }\frac{\partial ^{2}\Psi _{\kappa }(\chi ,\tau )}{\partial \chi ^{2}} -\zeta \sum _{\kappa =0}^{\infty }\Psi _{\kappa }(\chi ,\tau )+\zeta \sum _{\kappa =0}^{\infty } {\textbf {B}}_{\kappa }(\chi ,\tau )\right) \right] . \end{aligned} \end{aligned}$$

Furthermore, we acquire the following recursive equations from (64):

65 $$\begin{aligned} \begin{aligned}&\Phi _{0}^{{\Xi (\mu )}}(\chi ,\tau )=\frac{1}{\left( e^{\sqrt{\frac{\zeta }{6}}\chi }+1\right) ^{2}},\\&\Psi _{0}^{{\Xi (\mu )}}(\chi ,\tau )=\frac{\zeta -1}{\xi \left( e^{\sqrt{\frac{\zeta }{6}}\chi }+1\right) ^{2}},\\&\Phi _{\kappa +1}^{{\Xi (\mu )}}(\chi ,\tau )=-\mathbb{N}\mathbb{T}^{-1}\left[ {\textbf {F}}(\mu ,\omega ,s)\mathbb{N}\mathbb{T}^{+}\left[ -\frac{\partial ^{2}\Phi _{\kappa }(\chi ,\tau )}{\partial \chi ^{2}}-\xi \Psi _{\kappa }(\chi ,\tau )-\Phi _{\kappa }(\chi ,\tau )+{\textbf {A}}_{\kappa }(\chi ,\tau )+\xi {\textbf {B}}_{\kappa }(\chi ,\tau )\right] \right] ,\\&\Psi _{\kappa +1}^{{\Xi (\mu )}}(\chi ,\tau )=-\mathbb{N}\mathbb{T}^{-1}\left[ {\textbf {F}}(\mu ,\omega ,s)\mathbb{N}\mathbb{T}^{+}\left[ -\frac{\partial ^{2}\Psi _{\kappa }(\chi ,\tau )}{\partial \chi ^{2}}-\zeta \Psi _{\kappa }(\chi ,\tau )+\zeta {\textbf {B}}_{\kappa }(\chi ,\tau )\right] \right] , \ \ \ \kappa \ge 1. \end{aligned} \end{aligned}$$

#### NTDM series solution for (62) in Caputo sense

In view of (65) with

$${\textbf {F}}(\mu ,\omega ,s)=\left( \frac{\omega }{s}\right) ^{\mu },$$

we generate the following few iterations

$$\begin{aligned} \begin{aligned} \Phi ^{\texttt{C}}_{0}(\chi ,\tau )=&\frac{1}{\left( e^{\sqrt{\frac{\zeta }{6}}\chi }+1\right) ^{2}},\qquad & \Psi ^{\texttt{C}}_{0}(\chi ,\tau )=\frac{\zeta -1}{\xi \left( e^{\sqrt{\frac{\zeta }{6}}\chi }+1\right) ^{2}},\\ \Phi _1^{\texttt{C}}(\chi ,\tau )=&\frac{5 \zeta \tau ^{\mu } e^{\sqrt{\frac{\zeta }{6}}\chi }}{3 \Gamma (\mu +1) \left( e^{\sqrt{\frac{\zeta }{6}}\chi }+1\right) ^3},\qquad & \Psi _1^{\texttt{C}}(\chi ,\tau )=\frac{5 (\zeta -1) \zeta \tau ^{\mu } e^{\sqrt{\frac{\zeta }{6}}\chi }}{3 \xi \Gamma (\mu +1) \left( e^{\sqrt{\frac{\zeta }{6}}\chi }+1\right) ^3},\\ \Phi _2^{\texttt{C}}(\chi ,\tau )=&\frac{25 \zeta ^2 \tau ^{2\mu } e^{\sqrt{\frac{\zeta }{6}}\chi } \left( 2 e^{\sqrt{\frac{\zeta }{6}}\chi }-1\right) }{18 \Gamma (2 \mu +1) \left( e^{\sqrt{\frac{\zeta }{6}}\chi }+1\right) ^4},\qquad & \Psi _2^{\texttt{C}}(\chi ,\tau )=\frac{25 (\zeta -1) \zeta ^2 \tau ^{2\mu } e^{\sqrt{\frac{\zeta }{6}}\chi } \left( 2 e^{\sqrt{\frac{\zeta }{6}}\chi }-1\right) }{18 \xi \Gamma (2 \mu +1) \left( e^{\sqrt{\frac{\zeta }{6}}\chi }+1\right) ^4}. \end{aligned} \end{aligned}$$

Similar expressions for $\Phi ^{\texttt{C}}_{\kappa }(\chi ,\tau )$ and $\Psi ^{\texttt{C}}_{\kappa }(\chi ,\tau )$ for $\kappa \ge 3$ can also be obtained using (65). Furthermore, the NTDM series solution for (62) in Caputo sense is given according to

66 $$\begin{aligned} \begin{aligned} \Phi ^{\texttt{C}}(\chi ,\tau )=&\Phi ^{\texttt{C}}_{0}(\chi ,\tau )+\Phi ^{\texttt{C}}_{1}(\chi ,\tau )+\Phi ^{\texttt{C}}_{2}(\chi ,\tau )+\Phi ^{\texttt{C}}_{3}(\chi ,\tau )+\cdots ,\\ \Psi ^{\texttt{C}}(\chi ,\tau )=&\Psi ^{\texttt{C}}_{0}(\chi ,\tau )+\Psi ^{\texttt{C}}_{1}(\chi ,\tau )+\Psi ^{\texttt{C}}_{2}(\chi ,\tau )+\Psi ^{\texttt{C}}_{3}(\chi ,\tau )+\cdots . \end{aligned} \end{aligned}$$

#### NTDM series solution for (62) in $\texttt{CF}$ sense

In view of (65) with

$${\textbf {F}}(\mu ,\omega ,s)=1-\mu +\mu \left( \frac{\omega }{s}\right) ,$$

we obtain the following few iterations

$$\begin{aligned} \begin{aligned} \Phi ^{\texttt{CF}}_{0}(\chi ,\tau )=&\frac{1}{\left( e^{\sqrt{\frac{\zeta }{6}}\chi }+1\right) ^{2}},\qquad \qquad \qquad \quad \Psi ^{\texttt{CF}}_{0}(\chi ,\tau )=\frac{\zeta -1}{\xi \left( e^{\sqrt{\frac{\zeta }{6}}\chi }+1\right) ^{2}},\\ \Phi _1^{\texttt{CF}}(\chi ,\tau )=&\frac{5 \zeta (\mu (\tau -1)+1) e^{\sqrt{\frac{\zeta }{6}}\chi }}{3 \left( e^{\sqrt{\frac{\zeta }{6}}\chi }+1\right) ^3},\qquad \qquad \Psi _1^{\texttt{CF}}(\chi ,\tau )=\frac{5 \zeta (\mu (\tau -1)+1) e^{\sqrt{\frac{\zeta }{6}}\chi }}{3 \left( e^{\sqrt{\frac{\zeta }{6}}\chi }+1\right) ^3},\\ \Phi _2^{\texttt{CF}}(\chi ,\tau )=&\frac{25 \zeta ^2 \left( \mu ^2 \left( \tau ^2-4 \tau +2\right) +4\mu (\tau -1)+2\right) e^{\sqrt{\frac{\zeta }{6}}\chi } \left( 2 e^{\sqrt{\frac{\zeta }{6}}\chi }-1\right) }{36 \left( e^{\sqrt{\frac{\zeta }{6}}\chi }+1\right) ^4},\\ \Psi _2^{\texttt{CF}}(\chi ,\tau )=&\frac{25 (\zeta -1) \zeta ^2 \left( \mu ^2 ((\tau -4) \tau +2)+4\mu (\tau -1)+2\right) e^{\sqrt{\frac{\zeta }{6}}\chi } \left( 2 e^{\sqrt{\frac{\zeta }{6}}\chi }-1\right) }{36 \xi \left( e^{\sqrt{\frac{\zeta }{6}}\chi }+1\right) ^4}. \end{aligned} \end{aligned}$$

Similar expressions for $\Phi ^{\texttt{CF}}_{\kappa }(\chi ,\tau )$ and $\Psi ^{\texttt{CF}}_{\kappa }(\chi ,\tau )$ for $\kappa \ge 3$ can also be acquired using (65). Furthermore, the NTDM series solution for (62) with $\texttt{CF}$ derivative is given according to

67 $$\begin{aligned} \begin{aligned} \Phi ^{\texttt{CF}}(\chi ,\tau )=&\Phi ^{\texttt{CF}}_{0}(\chi ,\tau )+\Phi ^{\texttt{CF}}_{1}(\chi ,\tau )+\Phi ^{\texttt{CF}}_{2}(\chi ,\tau )+\Phi ^{\texttt{CF}}_{3}(\chi ,\tau )+\cdots ,\\ \Psi ^{\texttt{CF}}(\chi ,\tau )=&\Psi ^{\texttt{CF}}_{0}(\chi ,\tau )+\Psi ^{\texttt{CF}}_{1}(\chi ,\tau )+\Psi ^{\texttt{CF}}_{2}(\chi ,\tau )+\Psi ^{\texttt{CF}}_{3}(\chi ,\tau )+\cdots . \end{aligned} \end{aligned}$$

#### NTDM series solution for (62) in $\texttt{AB}$ sense

In view of (65) with $\mathcal {B}(\mu )=1$ and

$${\textbf {F}}(\mu ,\omega ,s)=1-\mu +\mu \left( \frac{\omega }{s}\right) ^{\mu },$$

we obtain the following few iterations

$$\begin{aligned} \begin{aligned} \Phi ^{\texttt{AB}}_{0}(\chi ,\tau )=&\frac{1}{\left( e^{\sqrt{\frac{\zeta }{6}}\chi }+1\right) ^{2}},\qquad \qquad \qquad \qquad \qquad \Psi ^{\texttt{AB}}_{0}(\chi ,\tau )=\frac{\zeta -1}{\xi \left( e^{\sqrt{\frac{\zeta }{6}}\chi }+1\right) ^{2}},\\ \Phi _1^{\texttt{AB}}(\chi ,\tau )=&\frac{5 \zeta e^{\sqrt{\frac{\zeta }{6}}\chi } \left( \mu \tau ^{\mu }-(\mu -1) \Gamma (\mu +1)\right) }{3 \Gamma (\mu +1) \left( e^{\sqrt{\frac{\zeta }{6}}\chi }+1\right) ^3},\qquad \Psi _1^{\texttt{AB}}(\chi ,\tau )=\frac{5 (\zeta -1) \zeta e^{\sqrt{\frac{\zeta }{6}}\chi } \left( \mu \tau ^{\mu }-(\mu -1) \Gamma (\mu +1)\right) }{3 \xi \Gamma (\mu +1) \left( e^{\sqrt{\frac{\zeta }{6}}\chi }+1\right) ^3},\\ \Phi _2^{\texttt{AB}}(\chi ,\tau )=&\frac{25 \zeta ^2 e^{\sqrt{\frac{\zeta }{6}}\chi } \left( 2 e^{\sqrt{\frac{\zeta }{6}}\chi }-1\right) \left( \Gamma (\mu +1) \left( (\mu -1)^2 \Gamma (2 \mu +1)+\mu ^2 \tau ^{2\mu }\right) -2 (\mu -1) \mu \Gamma (2 \mu +1) \tau ^{\mu }\right) }{18 \Gamma (\mu +1) \Gamma (2 \mu +1) \left( e^{\sqrt{\frac{\zeta }{6}}\chi }+1\right) ^4},\\ \Psi _2^{\texttt{AB}}(\chi ,\tau )=&\frac{25 (\zeta -1) \zeta ^2 e^{\sqrt{\frac{\zeta }{6}}\chi } \left( 2 e^{\sqrt{\frac{\zeta }{6}}\chi }-1\right) \left( \Gamma (\mu +1) \left( (\mu -1)^2 \Gamma (2 \mu +1)+\mu ^2 \tau ^{2\mu }\right) -2 (\mu -1) \mu \Gamma (2 \mu +1) \tau ^{\mu }\right) }{18 \xi \Gamma (\mu +1) \Gamma (2 \mu +1) \left( e^{\sqrt{\frac{\zeta }{6}}\chi }+1\right) ^4}. \end{aligned} \end{aligned}$$

Similar expressions for $\Phi ^{\texttt{AB}}_{\kappa }(\chi ,\tau )$ and $\Psi ^{\texttt{AB}}_{\kappa }(\chi ,\tau )$ for $\kappa \ge 3$ can also be acquired using (65). Furthermore, the NTDM series solution for (62) with $\texttt{AB}$ derivative is given according to

68 $$\begin{aligned} \begin{aligned} \Phi ^{\texttt{AB}}(\chi ,\tau )=&\Phi ^{\texttt{AB}}_{0}(\chi ,\tau )+\Phi ^{\texttt{AB}}_{1}(\chi ,\tau )+\Phi ^{\texttt{AB}}_{2}(\chi ,\tau )+\Phi ^{\texttt{AB}}_{3}(\chi ,\tau )+\cdots ,\\ \Psi ^{\texttt{AB}}(\chi ,\tau )=&\Psi ^{\texttt{AB}}_{0}(\chi ,\tau )+\Psi ^{\texttt{AB}}_{1}(\chi ,\tau )+\Psi ^{\texttt{AB}}_{2}(\chi ,\tau )+\Psi ^{\texttt{AB}}_{3}(\chi ,\tau )+\cdots . \end{aligned} \end{aligned}$$

## Discussion of results

This section presents a discussion of results obtained via the NTDM for the approximate solutions of the TF-BZ model considered within the scope of Caputo, $\texttt{CF}$ and $\texttt{AB}$ derivatives. In demonstrating the behaviors of the chemical wave profiles for the concentrations of the intermediates $\Phi (\chi ,\tau )$ and $\Psi (\chi ,\tau )$ with respect to varying parameter values, these results are presented in 2D and 3D graphical representations as well as tabular displays showing comparisons of numerical errors for various values of the spatial and temporal variables at $\mu =1$. For $\xi =2$, $\zeta =3$ and $\mu =1$, Tables 1, 2 show comparisons of absolute errors obtained for the approximate solutions of $\Phi (\chi ,\tau )$ and $\Psi (\chi ,\tau )$ in **Problem 1** with respect to the Caputo, $\texttt{CF}$ and $\texttt{AB}$ derivatives. Tables 3, 4 display comparisons of absolute errors obtained for the approximate solutions of $\Phi (\chi ,\tau )$ and $\Psi (\chi ,\tau )$ in **Problem 2** with respect to the Caputo, $\texttt{CF}$ and $\texttt{AB}$ derivatives when $\xi =2$, $\zeta =2$ and $\mu =1$. As presented in these tables the considered method demonstrates good accuracy as the results closely approximate those obtained via FRDTM^34^, $q-$HATM^34^, NTIM^41^ and OHAM^41^ for the Caputo derivative. Tables 5 and 6 present numerical results obtained for **Problem 1** and **Problem 2**, respectively, for different values of $\chi$ and $\tau$ and for $\mu =0.25, 0.5, 0.75, 1$. These numerical solutions are obtained for TF-BZ model in **Problem 1** and **Problem 2** with respect to the Caputo, $\texttt{CF}$ and $\texttt{AB}$ derivatives and are also compared with the exact solutions. The results obtained via the considered method demonstrate close proximity with that of the exact solutions for any given values of $\chi$ and $\tau$. In particular, the results show that as the fractional index approaches 1, the approximate solutions with respect to all three derivatives approach the exact solutions.

**Table 1:** Comparison of absolute errors for $\Phi (\chi ,\tau )$ and $\Psi (\chi ,\tau )$ in **Problem 1** when $\xi =2$, $\zeta =3$, $\mu =1$.

$\chi$	$\tau$	$|\Phi ^{\texttt{Exact}}-\Phi ^{\texttt{C}}|$	$|\Phi ^{\texttt{Exact}}-\Phi ^{\texttt{CF}}|$	$|\Phi ^{\texttt{Exact}}-\Phi ^{\texttt{AB}}|$	FRDTM^34^	q-HATM^34^
0	0.01	2.07041$e-11$	2.07016$e-11$	2.07041$e-11$	2.07040$e-11$	2.07040$e-11$
	0.03	5.20348$e-09$	5.20348$e-09$	5.20348$e-09$	5.20348$e-09$	5.20348$e-09$
	0.05	6.90958$e-08$	6.90958$e-08$	6.90958$e-08$	6.90958$e-08$	6.90958$e-08$
1	0.01	2.83274$e-11$	2.83278$e-11$	2.83274$e-11$	2.83274$e-11$	2.83274$e-11$
	0.03	6.84094$e-09$	6.84094$e-09$	6.84094$e-09$	6.84094$e-09$	6.84094$e-09$
	0.05	8.73606$e-08$	8.73606$e-08$	8.73606$e-08$	8.73606$e-08$	8.73606$e-08$
2	0.01	1.34195$e-11$	1.34164$e-11$	1.34195$e-11$	1.34195$e-11$	1.34195$e-11$
	0.03	3.33523$e-09$	3.33523$e-09$	3.33523$e-09$	3.33523$e-09$	3.33523$e-09$
	0.05	4.38521$e-08$	4.38521$e-08$	4.38521$e-08$	4.38521$e-08$	4.38521$e-08$
3	0.01	2.80693$e-12$	2.80955$e-12$	2.80693$e-12$	2.80693$e-12$	2.80693$e-12$
	0.03	6.64710$e-10$	6.64715$e-10$	6.64710$e-10$	6.64710$e-10$	6.64710$e-10$
	0.05	8.31173$e-09$	8.31173$e-09$	8.31173$e-09$	8.31173$e-09$	8.31173$e-09$
4	0.01	3.37710$e-12$	3.37825$e-12$	3.37710$e-12$	3.37710$e-12$	3.37710 $e-12$
	0.03	8.26454$e-10$	8.26457$e-10$	8.26454$e-10$	8.26454$e-10$	8.26454 $e-10$
	0.05	1.07019$e-08$	1.07019$e-08$	1.07019$e-08$	1.07019$e-08$	1.07019 $e-08$

$\chi$	$\tau$	$|\Psi ^{\texttt{Exact}}-\Psi ^{\texttt{C}}|$	$|\Psi ^{\texttt{Exact}}-\Psi ^{\texttt{CF}}|$	$|\Psi ^{\texttt{Exact}}-\Psi ^{\texttt{AB}}|$	FRDTM^34^	q-HATM^34^
0	0.01	2.07039$e-11$	2.07045$e-11$	2.07039$e-11$	2.07040$e-11$	2.07040$e-11$
	0.03	5.20348$e-09$	5.20348$e-09$	5.20348$e-09$	5.20348$e-09$	5.20348$e-09$
	0.05	6.90958$e-08$	6.90958$e-08$	6.90958$e-08$	6.90958$e-08$	6.90958$e-08$
1	0.01	2.83275$e-11$	2.83267$e-11$	2.83275$e-11$	2.83272$e-11$	2.83272$e-11$
	0.03	6.84094$e-09$	6.84094$e-09$	6.84094$e-09$	6.84094$e-09$	6.84094$e-09$
	0.05	8.73606$e-08$	8.73606$e-08$	8.73606$e-08$	8.73606$e-08$	8.73606$e-08$
2	0.01	1.34194$e-11$	1.34187$e-11$	1.34194$e-11$	1.34193$e-11$	1.34193$e-11$
	0.03	3.33523$e-09$	3.33523$e-09$	3.33523$e-09$	3.33523$e-09$	3.33523$e-09$
	0.05	4.38521$e-08$	4.38521$e-08$	4.38521$e-08$	4.38521$e-08$	4.38521$e-08$
	0.01	2.80698$e-12$	2.80720$e-12$	2.80698$e-12$	2.80687$e-12$	2.80687$e-12$
3	0.03	2.56908$e-10$	2.56869$e-10$	2.56908$e-10$	6.64710$e-10$	6.64710$e-10$
	0.05	8.31173$e-09$	8.31173$e-09$	8.31173$e-09$	8.31173$e-09$	8.31173$e-09$
	0.01	3.37697$e-12$	3.37708$e-12$	3.37697$e-12$	3.37730$e-12$	3.37730$e-12$
4	0.03	8.26454$e-10$	8.26454$e-10$	8.26454$e-10$	8.26455$e-10$	8.26455$e-10$
	0.05	1.07019$e-08$	1.07019$e-08$	1.07019$e-08$	1.07019$e-08$	1.07019$e-08$

**Table 2:** Comparison of absolute errors for $\Phi (\chi ,\tau )$ and $\Psi (\chi ,\tau )$ in **Problem 1** when $\xi =2$, $\zeta =3$, $\mu =1$.

$\chi$	$\tau$	$|\Phi ^{\texttt{Exact}}-\Phi ^{\texttt{C}}|$	$|\Phi ^{\texttt{Exact}}-\Phi ^{\texttt{CF}}|$	$|\Phi ^{\texttt{Exact}}-\Phi ^{\texttt{AB}}|$	NTIM^41^	OHAM^41^
	0.1	2.78454 $e-$10	2.78454 $e-$10	2.78454 $e-$10	3.46778 $e-$07	3.27824 $e-$05
0.001	0.3	1.7778$e-$10	1.7778$e-$10	1.7778$e-$10	3.79106$e-$07	3.00729$e-$05
	0.5	7.63204$e-$11	7.63204$e-$11	7.63204$e-$11	3.97046$e-$07	2.71800$e-$05
	0.1	7.54153$e-$09	7.54153$e-$09	7.54153$e-$09	3.11923$e-$06	9.56651$e-$05
0.003	0.3	4.8245$e-$09	4.8245$e-$09	4.8245$e-$09	3.41148$e-$06	8.76522$e-$05
	0.5	2.08434$e-$09	2.08434$e-$09	2.08434$e-$09	3.57426$e-$06	7.91159$e-$05
	0.1	3.5022$e-$08	3.5022$e-$08	3.5022$e-$08	8.65953$e-$06	1.54961$e-$04
0.005	0.3	2.24488$e-$08	2.24488$e-$08	2.24488$e-$08	9.47491$e-$06	1.41802$e-$04
	0.5	9.75955$e-$09	9.75955$e-$09	9.75955$e-$09	9.93078$e-$06	1.27816$e-$04
	0.1	9.6395$e-$08	9.6395$e-$08	9.6395$e-$08	1.69627$e-$05	2.10655$e-$04
0.007	0.3	6.19098$e-$08	6.19098$e-$08	6.19098$e-$08	1.85679$e-$05	1.92514$e-$04
	0.5	2.70818$e-$08	2.70818$e-$08	2.70818$e-$08	1.94686$e-$05	1.73277$e-$04

$\chi$	$\tau$	$|\Psi ^{\texttt{Exact}}-\Psi ^{\texttt{C}}|$	$|\Psi ^{\texttt{Exact}}-\Psi ^{\texttt{CF}}|$	$|\Psi ^{\texttt{Exact}}-\Psi ^{\texttt{AB}}|$	NTIM^41^	OHAM^41^
	0.1	2.78454$e-10$	2.78454$e-10$	2.78454$e-10$	3.46778$e-07$	1.18952$e-06$
0.001	0.3	1.7778$e-10$	1.7778$e-10$	1.7778$e-10$	3.79106$e-07$	1.13777$e-06$
	0.5	7.63203$e-11$	7.63203$e-11$	7.63203$e-11$	3.97046$e-07$	1.06865$e-06$
	0.1	7.54153$e-09$	7.54153$e-09$	7.54153$e-09$	3.11923$e-06$	7.39727$e-06$
0.003	0.3	4.8245$e-09$	4.8245$e-09$	4.8245$e-09$	3.41148$e-06$	7.20459$e-06$
	0.5	2.08434$e-09$	2.08434$e-09$	2.08434$e-09$	3.57426$e-06$	6.87421$e-06$
	0.1	3.5022$e-08$	3.5022$e-08$	3.5022$e-08$	8.65953$e-06$	1.86987$e-05$
0.005	0.3	2.24488$e-08$	2.24488$e-08$	2.24488$e-08$	9.47491$e-06$	1.83192$e-05$
	0.5	9.75955$e-09$	9.75955$e-09$	9.75955$e-09$	9.93078$e-06$	1.75676$e-05$
	0.1	9.6395$e-08$	9.6395$e-08$	9.6395$e-08$	1.69627$e-05$	3.50801$e-05$
0.007	0.3	6.19098$e-08$	6.19098$e-08$	6.19098$e-08$	1.85679$e-05$	3.44727$e-05$
	0.5	2.70818$e-08$	2.70818$e-08$	2.70818$e-08$	1.94686$e-05$	3.31448$e-05$

**Table 3:** Comparison of absolute errors for $\Phi (\chi ,\tau )$ and $\Psi (\chi ,\tau )$ in **Problem 2** when $\xi =2$, $\zeta =2$, $\mu =1$.

$\chi$	$\tau$	$|\Phi ^{\texttt{Exact}}-\Phi ^{\texttt{C}}|$	$|\Phi ^{\texttt{Exact}}-\Phi ^{\texttt{CF}}|$	$|\Phi ^{\texttt{Exact}}-\Phi ^{\texttt{AB}}|$	FRDTM^34^	q-HATM^34^
0	0.01	2.71078$e-12$	2.71033$e-12$	2.71078$e-12$	2.71072$e-12$	2.07040$e-12$
	0.03	6.73927$e-10$	6.73926$e-10$	6.73927$e-10$	6.73927$e-10$	6.73927$e-10$
	0.05	8.86033$e-09$	8.86033$e-09$	8.86033$e-09$	8.86033$e-09$	8.86033$e-09$
1	0.01	3.21043$e-12$	3.21060$e-12$	3.21043$e-12$	3.21052$e-12$	3.21051$e-12$
	0.03	7.72406$e-10$	7.72406$e-10$	7.72406$e-10$	7.72406$e-10$	7.72406$e-10$
	0.05	9.82970$e-09$	9.82970$e-09$	9.82970$e-09$	9.82970$e-09$	9.82970$e-09$
2	0.01	3.00746$e-12$	3.00693$e-12$	3.00746$e-12$	3.00748$e-12$	3.00748$e-12$
	0.03	7.36736$e-10$	7.36735$e-10$	7.36736$e-10$	7.36736$e-10$	7.36736$e-10$
	0.05	9.54952$e-09$	9.54953$e-09$	9.54952$e-09$	9.54952$e-09$	9.54952$e-09$
3	0.01	4.48808$e-13$	4.48114$e-13$	4.48808$e-13$	4.48808$e-13$	4.48808$e-13$
	0.03	1.13509$e-10$	1.13508$e-10$	1.13509$e-10$	1.13509$e-10$	1.13509$e-10$
	0.05	1.51797$e-09$	1.51797$e-09$	1.51797$e-09$	1.51797$e-09$	1.51797$e-09$
4	0.01	5.04097$e-13$	5.04558$e-13$	5.04097$e-13$	5.04100$e-13$	5.04100$e-13$
	0.03	1.21954$e-10$	1.21955$e-10$	1.21954$e-10$	1.21954$e-10$	1.21954$e-10$
	0.05	1.56086$e-09$	1.56086$e-09$	1.56086$e-09$	1.56086$e-09$	1.56086$e-09$

		$|\Psi ^{\texttt{Exact}}-\Psi ^{\texttt{C}}|$	$|\Psi ^{\texttt{Exact}}-\Psi ^{\texttt{CF}}|$	$|\Psi ^{\texttt{Exact}}-\Psi ^{\texttt{AB}}|$	FRDTM^34^	q-HATM^34^
0	0.01	1.35539$e-12$	1.35547$e-12$	1.35539$e-12$	1.35536$e-12$	1.35536$e-12$
	0.03	3.36964$e-10$	3.36964$e-10$	3.36964$e-10$	3.36964$e-10$	3.36964$e-10$
	0.05	4.43017$e-09$	4.43017$e-09$	4.43017$e-09$	4.43017$e-09$	4.43017$e-09$
1	0.01	1.60522$e-12$	1.60510$e-12$	1.60522$e-12$	1.60526$e-12$	1.60526$e-12$
	0.03	3.86203$e-10$	3.86203$e-10$	3.86203$e-10$	3.86203$e-10$	3.86203$e-10$
	0.05	4.91485$e-09$	4.91485$e-09$	4.91485$e-09$	4.91485$e-09$	4.91485$e-09$
2	0.01	1.50373$e-12$	1.50362$e-12$	1.50373$e-12$	1.50374$e-12$	1.50374$e-12$
	0.03	3.68368$e-10$	3.68368$e-10$	3.68368$e-10$	3.68368$e-10$	3.68368$e-10$
	0.05	4.77476$e-09$	4.77476$e-09$	4.77476$e-09$	4.77476$e-09$	4.77476$e-09$
3	0.01	2.24404$e-13$	2.24336$e-13$	2.24404$e-13$	2.24404$e-13$	2.24404$e-13$
	0.03	5.67546$e-11$	5.67546$e-11$	5.67546$e-11$	5.67546$e-11$	5.67546$e-11$
	0.05	7.58984$e-10$	7.58984$e-10$	7.58984$e-10$	7.58984$e-10$	7.58984$e-10$
4	0.01	2.52048$e-13$	2.52081$e-13$	2.52048$e-13$	2.52050$e-13$	2.52050$e-13$
	0.03	6.09771$e-11$	6.09771$e-11$	6.09771$e-11$	6.09771$e-11$	6.09771$e-11$
	0.05	7.80430$e-10$	7.80430$e-10$	7.80430$e-10$	7.80430$e-10$	7.80430$e-10$

**Table 4:** Comparison of absolute errors for $\Phi (\chi ,\tau )$ and $\Psi (\chi ,\tau )$ in **Problem 2** when $\xi =2$, $\zeta =2$, $\mu =1$.

$\chi$	$\tau$	$|\Phi ^{\texttt{Exact}}-\Phi ^{\texttt{C}}|$	$|\Phi ^{\texttt{Exact}}-\Phi ^{\texttt{CF}}|$	$|\Phi ^{\texttt{Exact}}-\Phi ^{\texttt{AB}}|$	NTIM^41^	OHAM^41^
0.001	0.1	8.51031 $e-$11	8.51031 $e-$11	8.51031 $e-$11	2.38719 $e-$11	2.88115$e-$06
	0.3	6.09674$e-$11	6.09674$e-$11	6.09674$e-$11	3.42036$e-$11	2.70417$e-$06
	0.5	3.62378$e-$11	3.62378$e-$11	3.62378$e-$11	4.52451$e-$11	2.51242$e-$06
0.003	0.1	2.30234$e-$09	2.30234$e-$09	2.30234$e-$09	6.39987$e-$10	1.02084$e-$05
	0.3	1.65092$e-$09	1.65092$e-$09	1.65092$e-$09	9.18695$e-$10	9.64541$e-$06
	0.5	9.83212$e-$10	9.83212$e-$10	9.83212$e-$10	1.21683$e-$09	9.0176$e-$06
0.005	0.1	1.06800$e-$08	1.06800$e-$08	1.06800$e-$08	2.94183$e-$09	1.9619$e-$05
	0.3	7.6654$e-$09	7.6654$e-$09	7.6654$e-$09	4.23099$e-$09	1.86281$e-$05
	0.5	4.57410$e-$09	4.57410$e-$09	4.57410$e-$09	5.61126$e-$09	1.74951$e-$05
0.007	0.1	2.93638$e-$08	2.93638$e-$08	2.93638$e-$08	8.01463$e-$09	3.11086$e-$05
	0.3	2.10948$e-$08	2.10948$e-$08	2.10948$e-$08	1.15488$e-$08	2.96491$e-$05
	0.5	1.26122$e-$08	1.26122$e-$08	1.26122$e-$08	1.53364$e-$08	2.79431$e-$05

$\chi$	$\tau$	$|\Psi ^{\texttt{Exact}}-\Psi ^{\texttt{C}}|$	$|\Psi ^{\texttt{Exact}}-\Psi ^{\texttt{CF}}|$	$|\Psi ^{\texttt{Exact}}-\Psi ^{\texttt{AB}}|$	NTIM^41^	OHAM^41^
0.001	0.1	4.25514$e-11$	4.25514$e-11$	4.25514$e-11$	1.1936$e-11$	8.90479$e-06$
	0.3	3.04836$e-11$	3.04836$e-11$	3.04836$e-11$	1.71018$e-11$	8.31684$e-06$
	0.5	1.81189$e-11$	1.81189$e-11$	1.81189$e-11$	2.26226$e-11$	7.69092$e-06$
0.003	0.1	1.15117$e-09$	1.15117$e-09$	1.15117$e-09$	3.19993$e-10$	2.62487$e-05$
	0.3	8.25461$e-10$	8.25461$e-10$	8.25461$e-10$	4.59347$e-10$	2.44863$e-05$
	0.5	4.91606$e-10$	4.91606$e-10$	4.91606$e-10$	6.08413$e-10$	2.26156$e-06$
0.005	0.1	5.34002$e-09$	5.34002$e-09$	5.34002$e-09$	1.47092$e-09$	4.29701$e-05$
	0.3	3.83270$e-09$	3.83270$e-09$	3.83270$e-09$	2.11549$e-09$	4.00354$e-05$
	0.5	2.28705$e-09$	2.28705$e-09$	2.28705$e-09$	2.80563$e-09$	3.69301$e-05$
0.007	0.1	1.46819$e-08$	1.46819$e-08$	1.46819$e-08$	4.00732$e-09$	5.90667$e-05$
	0.3	1.05474$e-08$	1.05474$e-08$	1.05474$e-08$	5.77442$e-09$	5.49628$e-05$
	0.5	6.30612$e-09$	6.30612$e-09$	6.30612$e-09$	7.6682$e-09$	5.06334$e-05$

**Table 5:** Numerical results for $\Phi (\chi ,\tau )$ and $\Psi (\chi ,\tau )$ in **Problem 1** with respect to Caputo, $\texttt{CF}$ and $\texttt{AB}$ derivatives and different values of $\mu$.

$\chi$	$\tau$	$\mu =0.25$			$\mu =0.5$			$\mu =0.75$			$\mu =1$			
		$\Phi ^{\texttt{C}}(\chi ,\tau )$	$\Phi ^{\texttt{CF}}(\chi ,\tau )$	$\Phi ^{\texttt{AB}}(\chi ,\tau )$	$\Phi ^{\texttt{C}}(\chi ,\tau )$	$\Phi ^{\texttt{CF}}(\chi ,\tau )$	$\Phi ^{\texttt{AB}}(\chi ,\tau )$	$\Phi ^{\texttt{C}}(\chi ,\tau )$	$\Phi ^{\texttt{CF}}(\chi ,\tau )$	$\Phi ^{\texttt{AB}}(\chi ,\tau )$	$\Phi ^{\texttt{C}}(\chi ,\tau )$	$\Phi ^{\texttt{CF}}(\chi ,\tau )$	$\Phi ^{\texttt{AB}}(\chi ,\tau )$	Exact
0	0.01	0.49636	0.04699	− 0.33421	0.32729	0.50549	0.47244	0.27207	0.43867	0.45131	0.25629	0.25629	0.25629	0.25629
	0.03	0.53178	0.02843	− 0.49065	0.38934	0.50083	0.4291	0.30183	0.44907	0.47258	0.26909	0.26909	0.26909	0.26909
	0.05	0.52374	0.0095	-0.58715	0.43211	0.4955	0.38948	0.32767	0.4591	0.48851	0.28218	0.28218	0.28218	0.28218
1	0.01	0.36338	0.98654	1.10328	0.16017	0.59615	0.67451	0.12233	0.27886	0.297	0.11276	0.11276	0.11276	0.11276
	0.03	0.49639	0.99393	1.13589	0.21114	0.61122	0.73721	0.14143	0.29357	0.33074	0.12043	0.12043	0.12043	0.12043
	0.05	0.57952	1.00122	1.15294	0.25308	0.6262	0.77981	0.15919	0.30846	0.35975	0.12849	0.12849	0.12849	0.12848
2	0.01	0.23224	1.99389	2.78488	0.0623	0.63036	0.81349	0.04397	0.1521	0.16937	0.03981	0.03981	0.03981	0.03981
	0.03	0.41410	2.03510	3.08512	0.09162	0.6632	0.98328	0.05267	0.16597	0.20353	0.04311	0.04311	0.04311	0.04311
	0.05	0.56328	2.07661	3.26549	0.11996	0.69671	1.11284	0.06125	0.18044	0.23508	0.04665	0.04665	0.04665	0.04665
3	0.01	0.10794	1.36783	1.97655	0.02012	0.36926	0.49644	0.01339	0.0644	0.07387	0.01198	0.01198	0.01198	0.01198
	0.03	0.21988	1.39918	2.21076	0.03225	0.39181	0.61678	0.01644	0.07197	0.09303	0.01309	0.01309	0.01309	0.01309
	0.05	0.31780	1.43081	2.35214	0.04520	0.41491	0.70988	0.01958	0.07996	0.11119	0.0143	0.01430	0.01430	0.01430
4	0.01	0.03797	0.56269	0.82203	0.00573	0.14370	0.19613	0.00367	0.02165	0.02522	0.00326	0.00326	0.00326	0.00326
	0.03	0.08210	0.57600	0.92221	0.00970	0.15296	0.24607	0.00458	0.02450	0.03252	0.00359	0.00359	0.00359	0.00359
	0.05	0.12162	0.58943	0.98278	0.01416	0.16246	0.28486	0.00553	0.02752	0.03950	0.00394	0.00394	0.00394	0.00394

$\chi$	$\tau$	$\mu =0.25$			$\mu =0.5$			$\mu =0.75$			$\mu =1$			
		$\Psi ^{\texttt{C}}(\chi ,\tau )$	$\Psi ^{\texttt{CF}}(\chi ,\tau )$	$\Psi ^{\texttt{AB}}(\chi ,\tau )$	$\Psi ^{\texttt{C}}(\chi ,\tau )$	$\Psi ^{\texttt{CF}}(\chi ,\tau )$	$\Psi ^{\texttt{AB}}(\chi ,\tau )$	$\Psi ^{\texttt{C}}(\chi ,\tau )$	$\Psi ^{\texttt{CF}}(\chi ,\tau )$	$\Psi ^{\texttt{AB}}(\chi ,\tau )$	$\Psi ^{\texttt{C}}(\chi ,\tau )$	$\Psi ^{\texttt{CF}}(\chi ,\tau )$	$\Psi ^{\texttt{AB}}(\chi ,\tau )$	
0	0.01	−0.50364	−0.95301	−1.33421	−0.67271	−0.49451	−0.52756	−0.72793	−0.56133	−0.54869	−0.74371	−0.74371	−0.74371	−0.74371
	0.03	−0.46822	−0.97157	−1.49065	−0.61066	−0.49917	−0.5709	−0.69817	−0.55093	−0.52742	−0.73091	−0.73091	−0.73091	−0.73091
	0.05	−0.47626	−0.99050	−1.58715	−0.56789	−0.50450	−0.61052	−0.67233	−0.54090	−0.51149	−0.71782	−0.71782	−0.71782	−0.71782
1	0.01	−0.63662	−0.01346	0.10328	−0.83983	−0.40385	−0.32549	−0.87767	−0.72114	−0.70300	−0.88724	−0.88724	−0.88724	−0.88724
	0.03	−0.50361	−0.00607	0.13589	−0.78886	−0.38878	−0.26279	−0.85857	−0.70643	−0.66926	−0.87957	−0.87957	−0.87957	−0.87957
	0.05	−0.42048	0.00122	0.15294	−0.74692	−0.3738	−0.22019	−0.84081	−0.69154	−0.64025	0.87151	−0.87151	−0.87151	−0.87152
2	0.01	−0.76776	0.99389	1.78488	−0.9377	−0.36964	−0.18651	−0.95603	−0.8479	−0.83063	−0.96019	−0.96019	−0.96019	−0.96019
	0.03	−0.58590	1.03510	2.08512	−0.90838	−0.3368	−0.01672	−0.94733	−0.83403	−0.79647	−0.95689	−0.95689	−0.95689	−0.95689
	0.05	−0.43672	1.07661	2.26549	−0.88004	−0.30329	0.11284	−0.93875	−0.81956	−0.76492	−0.95335	−0.95335	−0.95335	−0.95335
3	0.01	−0.89206	0.36783	0.97655	−0.97988	−0.63074	−0.50356	−0.98661	−0.9356	−0.92613	−0.98802	−0.98802	−0.98802	−0.98802
	0.03	−0.78012	0.39918	1.21076	−0.96775	−0.60819	−0.38322	−0.98356	−0.92803	−0.90697	−0.98691	−0.98691	−0.98691	−0.98691
	0.05	−0.68220	0.43081	1.35214	−0.9548	−0.58509	−0.29012	−0.98042	−0.92004	−0.88881	−0.98570	−0.98570	−0.98570	−0.98570
4	0.01	−0.96203	−0.43731	−0.17797	−0.99427	−0.8563	−0.80387	−0.99633	−0.97835	−0.97478	−0.99674	−0.99674	−0.99674	−0.99674
	0.03	−0.91790	−0.42400	−0.07779	−0.99030	−0.84704	−0.75393	−0.99542	−0.97550	−0.96748	−0.99641	−0.99641	−0.99641	−0.99641
	0.05	−0.87838	−0.41057	−0.01722	−0.98584	−0.83754	−0.71514	−0.99447	−0.97248	−0.96050	−0.99606	−0.99606	−0.99606	−0.99606

**Table 6:** Numerical results for $\Phi (\chi ,\tau )$ and $\Psi (\chi ,\tau )$ in **Problem 2** with respect to Caputo, $\texttt{CF}$ and $\texttt{AB}$ derivatives and different values of $\mu$.

$\chi$	$\tau$	$\mu =0.25$			$\mu =0.5$			$\mu =0.75$			$\mu =1$			
		$\Phi ^{\texttt{C}}(\chi ,\tau )$	$\Phi ^{\texttt{CF}}(\chi ,\tau )$	$\Phi ^{\texttt{AB}}(\chi ,\tau )$	$\Phi ^{\texttt{C}}(\chi ,\tau )$	$\Phi ^{\texttt{CF}}(\chi ,\tau )$	$\Phi ^{\texttt{AB}}(\chi ,\tau )$	$\Phi ^{\texttt{C}}(\chi ,\tau )$	$\Phi ^{\texttt{CF}}(\chi ,\tau )$	$\Phi ^{\texttt{AB}}(\chi ,\tau )$	$\Phi ^{\texttt{C}}(\chi ,\tau )$	$\Phi ^{\texttt{CF}}(\chi ,\tau )$	$\Phi ^{\texttt{AB}}(\chi ,\tau )$	$\Phi _{\texttt{Exact}}$
0	0.01	0.41976	0.50689	0.46996	0.3002	0.48779	0.50343	0.26459	0.37424	0.38365	0.25418	0.25418	0.25418	0.25418
	0.03	0.47122	0.50549	0.45153	0.34019	0.49116	0.51282	0.28397	0.38194	0.40044	0.26265	0.26265	0.26265	0.26265
	0.05	0.49579	0.50401	0.43954	0.36868	0.49439	0.51742	0.30063	0.3896	0.41417	0.27125	0.27125	0.27125	0.27125
1	0.01	0.2772	0.55736	0.60915	0.16479	0.38433	0.42008	0.13909	0.23122	0.24076	0.13205	0.13205	0.13205	0.13205
	0.03	0.34379	0.56062	0.62406	0.19649	0.39124	0.44875	0.1526	0.23899	0.25837	0.13774	0.13774	0.13774	0.13774
	0.05	0.38438	0.56384	0.632	0.22111	0.39812	0.46832	0.16465	0.24683	0.27338	0.14360	0.14360	0.14360	0.14360
2	0.01	0.16269	0.65324	0.8388	0.07732	0.29364	0.3479	0.06267	0.12518	0.13303	0.05889	0.05889	0.05889	0.05889
	0.03	0.23132	0.66318	0.90699	0.09713	0.30359	0.39625	0.07012	0.13153	0.14805	0.06193	0.06193	0.06193	0.06193
	0.05	0.28144	0.67317	0.94748	0.11390	0.31369	0.43214	0.07700	0.13806	0.16140	0.06510	0.06510	0.06510	0.06510
3	0.01	0.08226	0.51892	0.70908	0.03178	0.18449	0.23054	0.02493	0.059	0.06391	0.02325	0.02325	0.02325	0.02325
	0.03	0.13127	0.52887	0.78094	0.04175	0.19279	0.27294	0.02832	0.06296	0.07354	0.02459	0.02459	0.02459	0.02459
	0.05	0.17026	0.53889	0.82405	0.05081	0.20125	0.30516	0.03153	0.06707	0.08233	0.02601	0.02601	0.02601	0.02601
4	0.01	0.03579	0.29033	0.40696	0.01183	0.09214	0.11845	0.00907	0.02445	0.02686	0.00841	0.00841	0.00841	0.00841
	0.03	0.06161	0.29638	0.45144	0.01608	0.09685	0.14299	0.01041	0.02639	0.03166	0.00894	0.00894	0.00894	0.00894
	0.05	0.08300	0.30248	0.47821	0.02011	0.10165	0.16181	0.01171	0.02842	0.03610	0.00949	0.00949	0.00949	0.00949

		$\mu =0.25$			$\mu =0.5$			$\mu =0.75$			$\mu =1$			
		$\Psi ^{\texttt{C}}(\chi ,\tau )$	$\Psi ^{\texttt{CF}}(\chi ,\tau )$	$\Psi ^{\texttt{AB}}(\chi ,\tau )$	$\Psi ^{\texttt{C}}(\chi ,\tau )$	$\Psi ^{\texttt{CF}}(\chi ,\tau )$	$\Psi ^{\texttt{AB}}(\chi ,\tau )$	$\Psi ^{\texttt{C}}(\chi ,\tau )$	$\Psi ^{\texttt{CF}}(\chi ,\tau )$	$\Psi ^{\texttt{AB}}(\chi ,\tau )$	$\Psi ^{\texttt{C}}(\chi ,\tau )$	$\Psi ^{\texttt{CF}}(\chi ,\tau )$	$\Psi ^{\texttt{AB}}(\chi ,\tau )$	$\Psi _{\texttt{Exact}}$
0	0.01	0.20988	0.25345	0.23498	0.15010	0.24390	0.25171	0.13230	0.18712	0.19182	0.12709	0.12709	0.12709	0.12709
	0.03	0.23561	0.25274	0.22577	0.17010	0.24558	0.25641	0.14198	0.19097	0.20022	0.13133	0.13133	0.13133	0.13133
	0.05	0.24789	0.25200	0.21977	0.18434	0.24720	0.25871	0.15032	0.19480	0.20709	0.13563	0.13563	0.13563	0.13563
1	0.01	0.13860	0.27868	0.30458	0.08239	0.19216	0.21004	0.06954	0.11561	0.12038	0.06603	0.06603	0.06603	0.06603
	0.03	0.17190	0.28031	0.31203	0.09825	0.19562	0.22437	0.07630	0.11949	0.12918	0.06887	0.06887	0.06887	0.06887
	0.05	0.19219	0.28192	0.31600	0.11055	0.19906	0.23416	0.08232	0.12342	0.13669	0.07180	0.07180	0.07180	0.07180
2	0.01	0.08134	0.32662	0.41940	0.03866	0.14682	0.17395	0.03134	0.06259	0.06652	0.02945	0.02945	0.02945	0.02945
	0.03	0.11566	0.33159	0.45349	0.04856	0.15180	0.19813	0.03506	0.06577	0.07402	0.03097	0.03097	0.03097	0.03097
	0.05	0.14072	0.33658	0.47374	0.05695	0.15684	0.21607	0.03850	0.06903	0.08070	0.03255	0.03255	0.03255	0.03255
3	0.01	0.04113	0.25946	0.35454	0.01589	0.09225	0.11527	0.01246	0.02950	0.03196	0.01162	0.01162	0.01162	0.01162
	0.03	0.06563	0.26444	0.39047	0.02088	0.09640	0.13647	0.01416	0.03148	0.03677	0.01230	0.01230	0.01230	0.01230
	0.05	0.08513	0.26945	0.41202	0.02540	0.10063	0.15258	0.01577	0.03354	0.04117	0.01301	0.01301	0.01301	0.01301
4	0.01	0.01790	0.14516	0.20348	0.00592	0.04607	0.05922	0.00454	0.01222	0.01343	0.00421	0.00421	0.00421	0.00421
	0.03	0.03080	0.14819	0.22572	0.00804	0.04842	0.07150	0.00521	0.01319	0.01583	0.00447	0.00447	0.00447	0.00447
	0.05	0.04150	0.15124	0.23911	0.01006	0.05083	0.08090	0.00585	0.01421	0.01805	0.00475	0.00475	0.00475	0.00475

**Fig. 1:**
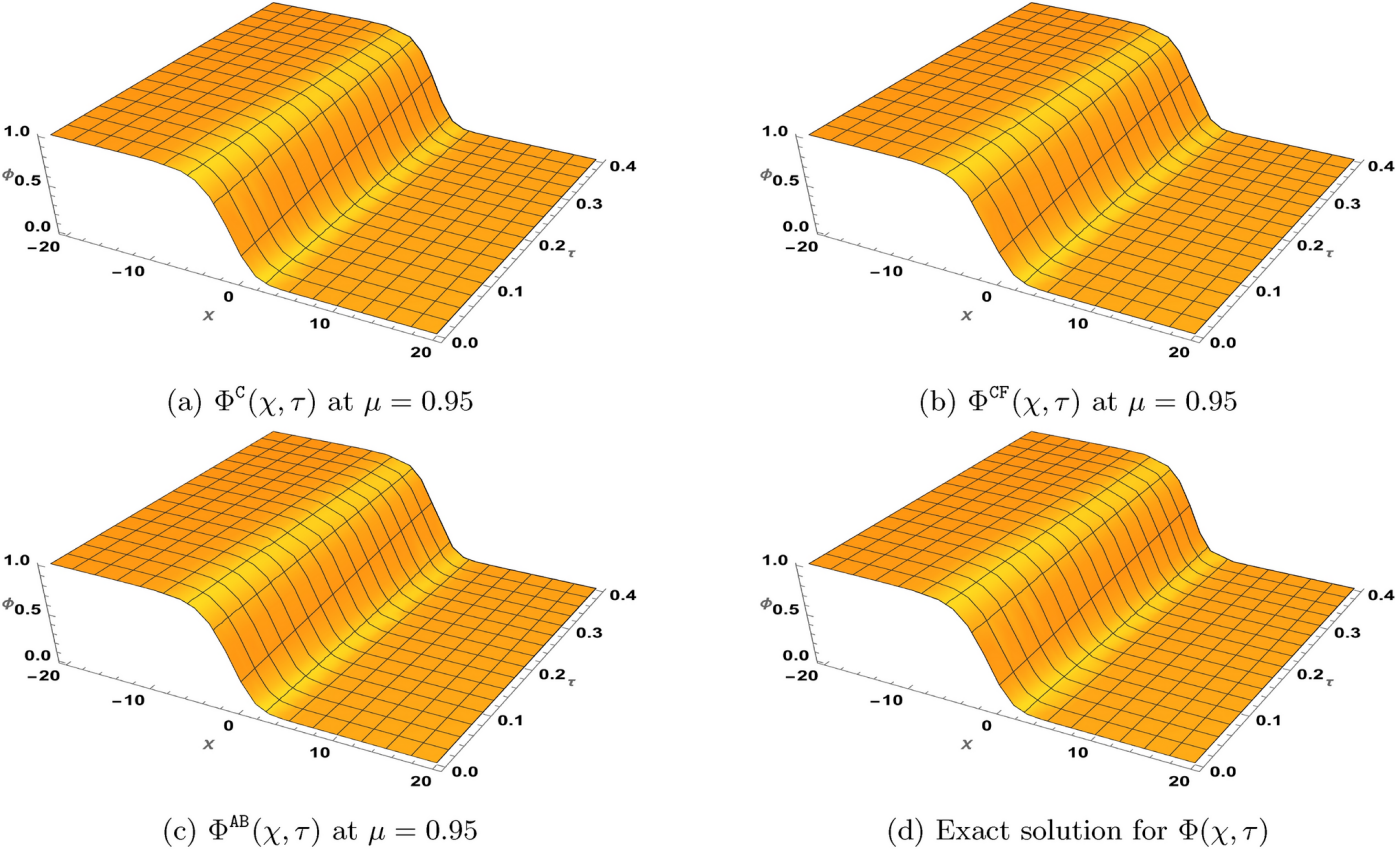
3D chemical wave oscillations of $\Phi (\chi ,\tau )$ in **Problem 1** at $\xi =2$ and $\zeta =3$.

**Fig. 2:**
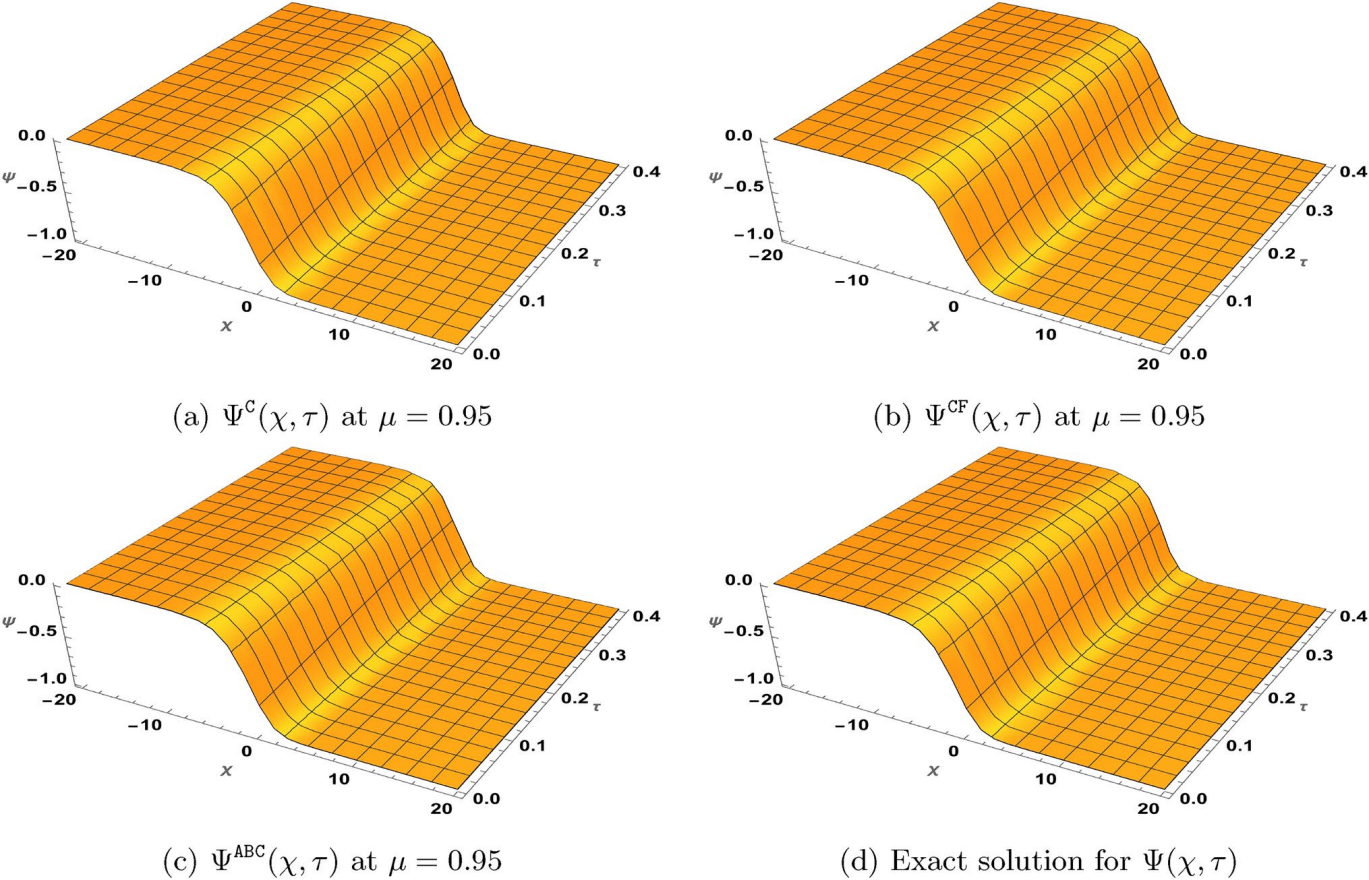
3D chemical wave oscillations of $\Psi (\chi ,\tau )$ in **Problem 1** at $\xi =2$ and $\zeta =3$.

**Fig. 3:**
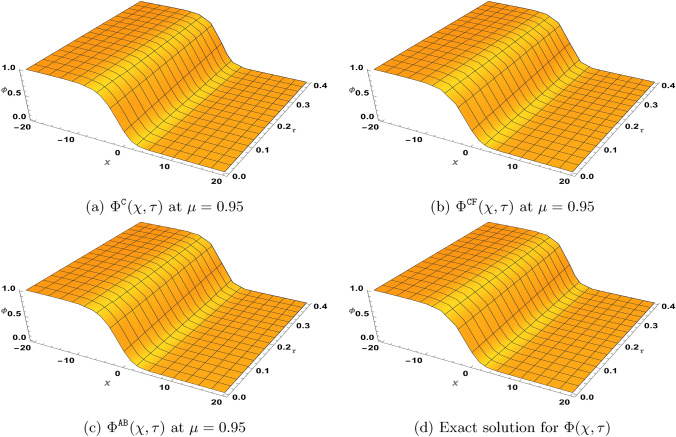
3D chemical wave oscillations of $\Phi (\chi ,\tau )$ in **Problem 2** at $\xi =2$ and $\zeta =2$.

**Fig. 4:**
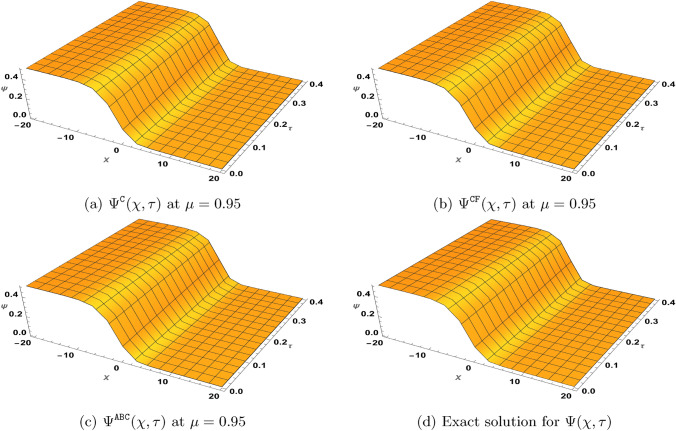
3D chemical wave oscillations of $\Psi (\chi ,\tau )$ in **Problem 2** at $\xi =2$ and $\zeta =2$.

**Fig. 5:**
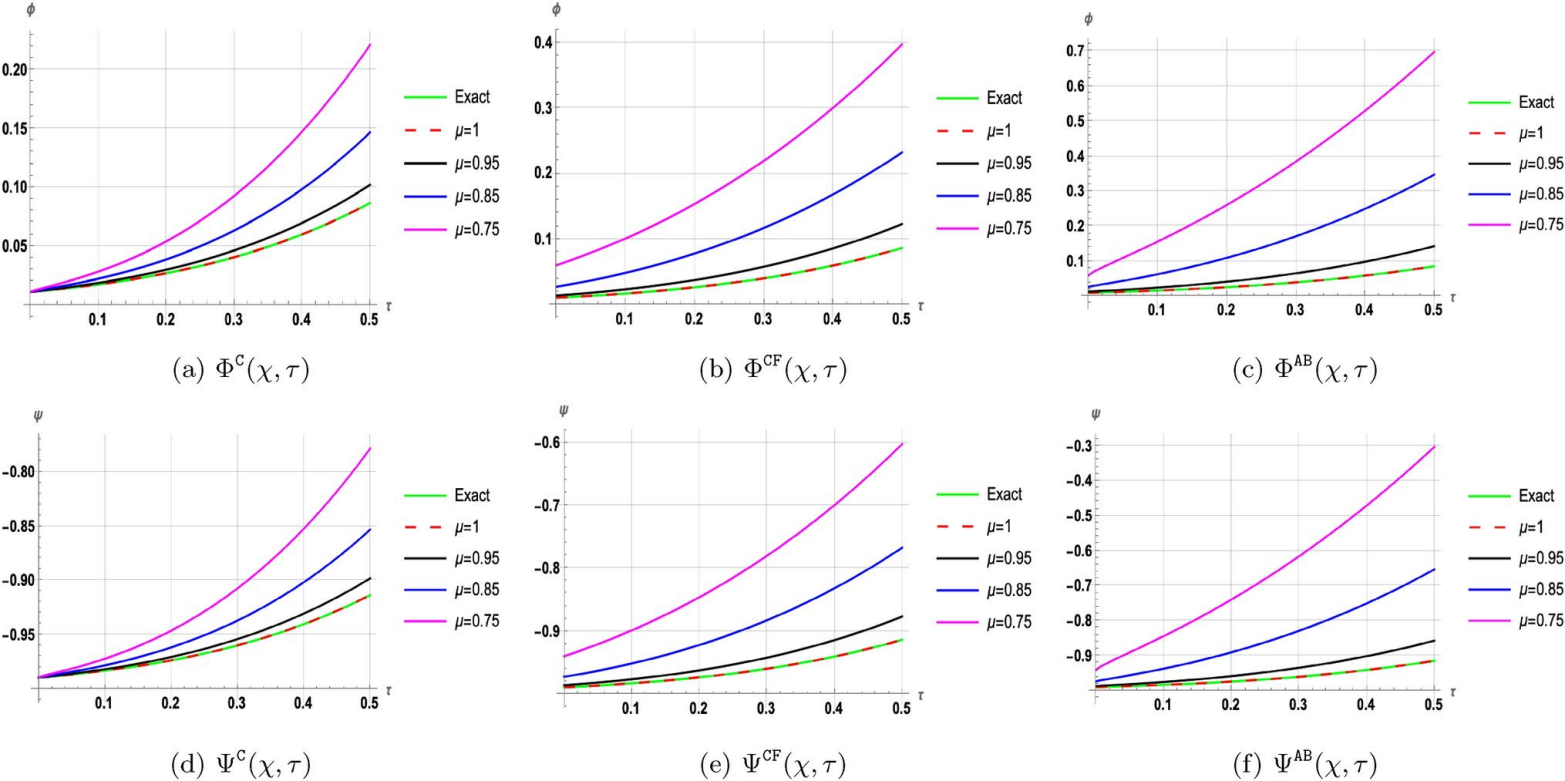
2D chemical wave profiles depicting solutions of $\Phi (\chi ,\tau )$ and $\Psi (\chi ,\tau )$ for **Problem 1** at $\chi =3$, $\xi =2$, $\zeta =3$.

**Fig. 6:**
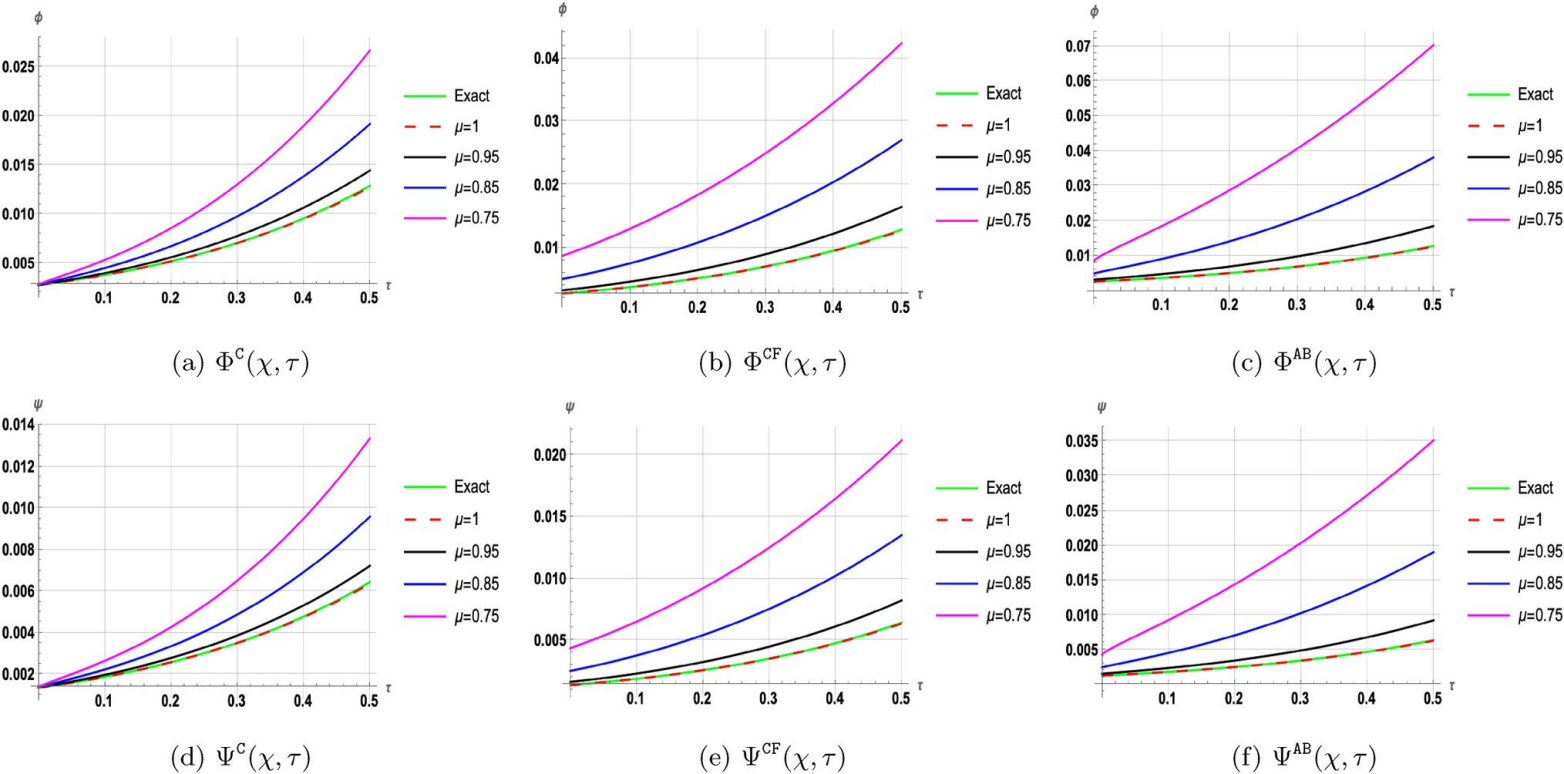
2D chemical wave profiles depicting solutions of $\Phi (\chi ,\tau )$ and $\Psi (\chi ,\tau )$ for **Problem 2** at $\chi =5$, $\xi =2$, $\zeta =2$.

**Fig. 7:**
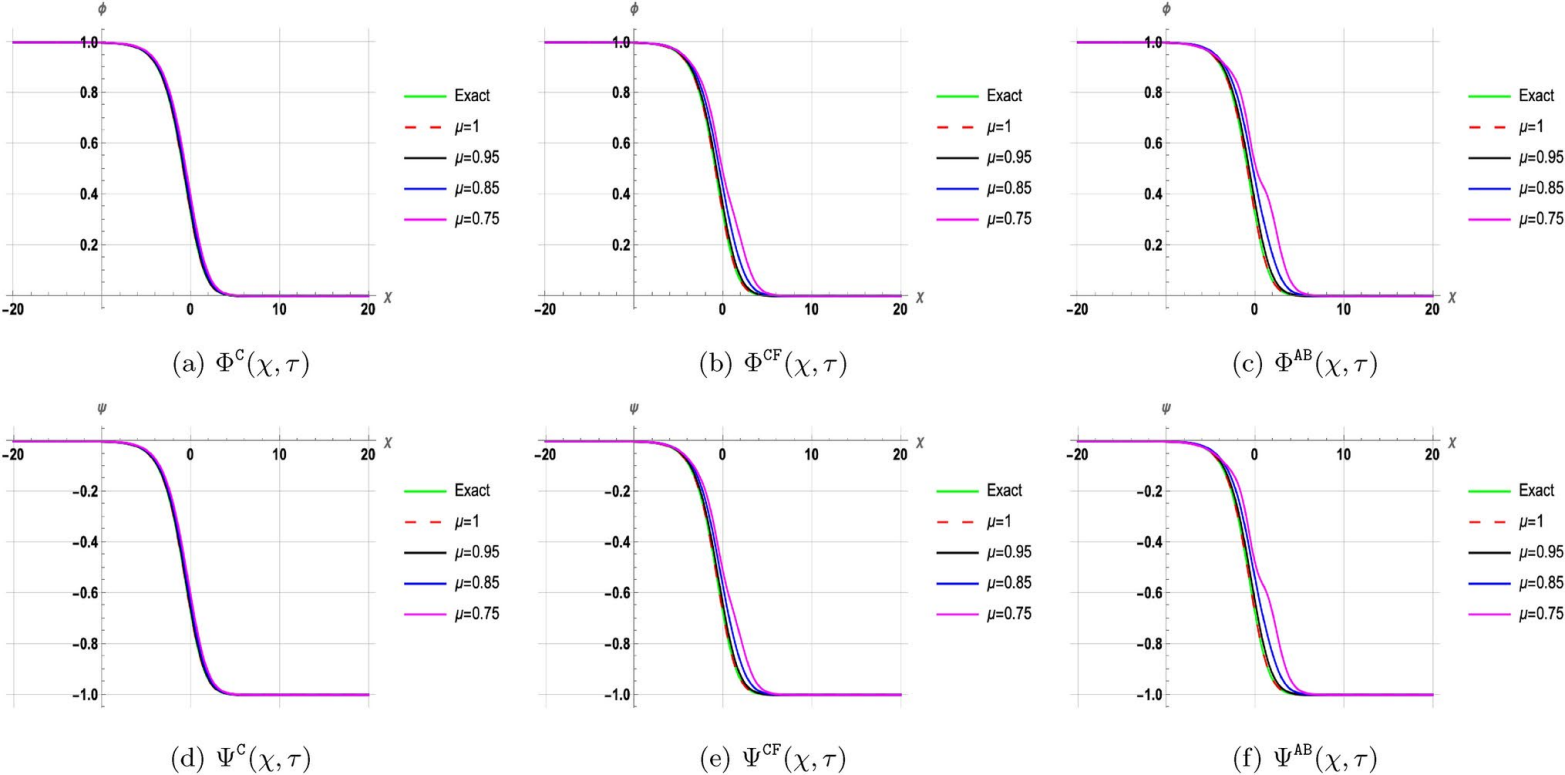
2D-plots depicting solutions for $\Phi (\chi ,\tau )$ and $\Psi (\chi ,\tau )$ of **Problem 1** at $\tau =0.1$, $\xi =2$, $\zeta =3$.

**Fig. 8:**
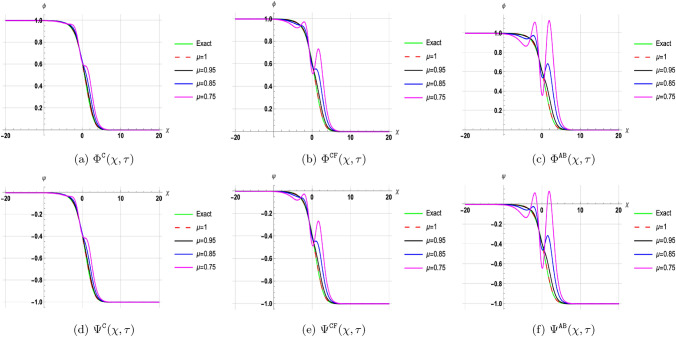
Solutions for $\Phi (\chi ,\tau )$ and $\Psi (\chi ,\tau )$ of **Problem 1** at $\tau =0.5$, $\xi =2$, $\zeta =3$.

**Fig. 9:**
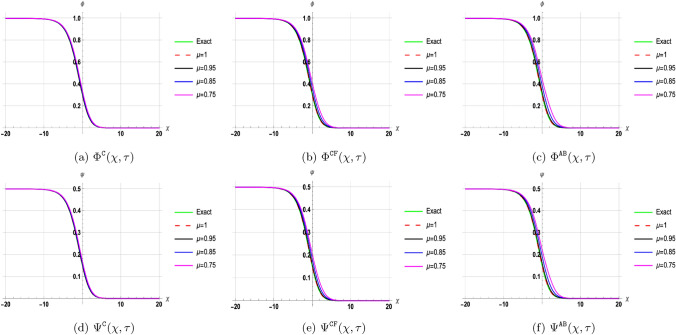
2D-plots depicting solutions for $\Phi (\chi ,\tau )$ and $\Psi (\chi ,\tau )$ of **Problem 2** at $\tau =0.1$, $\xi =2$ and $\zeta =2$.

**Fig. 10:**
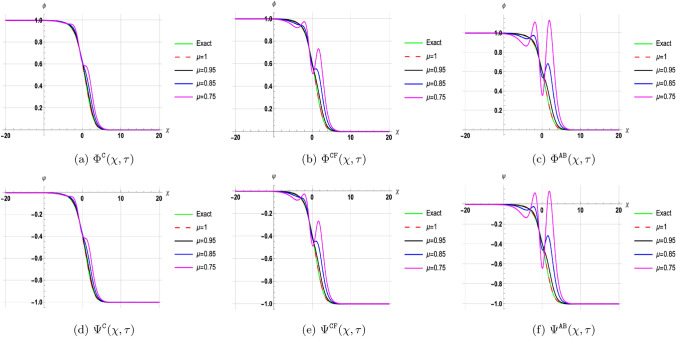
Solutions for $\Phi (\chi ,\tau )$ and $\Psi (\chi ,\tau )$ of **Problem 2** at $\tau =0.7$, $\xi =2$, $\zeta =2$.

Graphical simulations in 3D and 2D in Figs. 1, 2, 3, 4, 5, 6, 7, 8, 9 and 10 are presented to offer visual representations of the chemical wave profiles for **Problem 1** and **Problem 2**. This aim here is to provide insights into the systems dynamic behavior with respect to varying values of the fractional derivatives and for cases where $\chi$ and $\tau$ are considered fixed in the 2D plots. The 3D surface plots in Figs.  1 and 2 demonstrate the nature of the wave profiles when $\xi =2$ and $\zeta =3$ for the intermediates $\Phi (\chi ,\tau )$ and $\Psi (\chi ,\tau )$, respectively, in **Problem 1**. Similarly, by taking $\xi =2$ and $\zeta =2$, the 3D surface plots in Figs. 3 and 4 demonstrate the nature of the wave profiles for the intermediates $\Phi (\chi ,\tau )$ and $\Psi (\chi ,\tau )$, respectively, in **Problem 2**. It should be noted that in each of these figures, the wave profiles are shown for solutions obtained via the NDTM with respect to the Caputo, $\texttt{CF}$ and $\texttt{AB}$ fractional derivatives at $\mu =0.95$ as well as for the exact solutions. It is observed that for $\mu =0.95$, the 3D representations with respect to each of the considered fractional derivatives show similarity with the surface wave profiles of the exact solutions for each for the intermediates and for each version of the TF-BZ system. For fixed $\chi$, the 2D simulations in Figs.  5 and 6 depict the nature of the approximate solutions for **Problem 1** and **Problem 2**, respectively, with respect to the considered derivatives. In Figs. 5 we take $\chi =3$, $\xi =2$, $\zeta =3$ for **Problem 1** while in Fig. 6 we take $\chi =5$, $\xi =2$, $\zeta =2$ for **Problem 2**. These 2D plots demonstrate the influence of varying values of the fractional parameter index $\mu$ on the concentrations of the intermediates $\Phi (\chi ,\tau )$ and $\Psi (\chi ,\tau )$ for fixed $\chi$ and for $0\le \tau \le 0.5$. These simulations also show that as the value of $\mu$ increases from 0.75 to 1, the approximate solutions tend to the exact solutions. The plots in Figs. 7 and 8 are 2D wave profile representations of **Problem 1** for $\Phi (\chi ,\tau )$ and $\Psi (\chi ,\tau )$ at $\tau =0.1$ and $\tau =0.5$, respectively. Similarly, the plots in Figs. 9 and 10 are 2D wave profile representations of **Problem 2** for $\Phi (\chi ,\tau )$ and $\Psi (\chi ,\tau )$ at $\tau =0.1$ and $\tau =0.7$, respectively. For each problem, these wave profiles show the behavior of the concentrations of the intermediates under varying values of the fractional parameter index for $\mu (=0.75, 0.85, 0.95, 1)$ over the range $-20\le \chi \le 20$. These simulations demonstrate a some chaotic oscillatory trajectory at $\mu =0.75$ which are more obvious in Figs. 8 and 10 for the solutions of $\Psi (\chi ,\tau )$ where the wave curves exhibit some marked deviations from those of the exact solutions. However as $\mu$ approaches 1, these behaviors decreases so that the wave curves for the approximate solutions align with that of the exact solution. The behaviors in Figs. 7, 8, 9 and 10 assert that the concentrations of the intermediates attains uniform dynamics as $\mu \rightarrow 1$. These analysis and assertions provide evidence that the considered method yield approximate solutions that exhibit consistent behaviors for all three fractional derivatives. Furthermore, the obtained approximate solutions demonstrate good resemblance to the exact solutions.

## Conclusion

The time-fractional Belousov–Zhabotinsky system was solved using the natural transform decomposition method. To evaluate the efficacy of the method, we examined two test cases of the considered problem. In each of the considered cases, we examined the considered model within the context of the Caputo, CF, and AB fractional derivatives. The effect of the varying values of the fractional parameter index was observed and presented via comparison of approximate solutions as well as simulations in 2D and 3D plots demonstrating the dynamic behaviors of chemical wave profiles of the intermediates. The obtained numerical solutions and absolute errors with respect to each of the considered fractional derivatives are compared with the exact solutions as well as with those from related literature. The graphical representations provided more interesting physical behavior of the model in terms of varying values of the index of differentiation. Particularly, the simulations show the behavior of constituent chemicals of the Belousov–Zhabotinsky reaction system over temporal and spatial dimensions by using fractional derivatives concepts to capture non-local and memory-dependent features. This allow us to obtain more accurate representations of the complex spatio-temporal trajectories that can be observed in the reaction system. The results obtained here shown that the methodology employed is capable of yielding approximate solutions that exhibit good convergence towards the exact solutions as $\mu \rightarrow 1$. Hence, the current approach has the potential to serve as a valuable instrument for investigating various nonlinear time fractional systems of PDEs modeling diverse physical systems that do not attain thermodynamic equilibrium.

## Data Availability

The datasets used and analyzed during the current study available from the corresponding author on reasonable request.
